# MIND Dietary Pattern and Its Association with Cognition and Incident Dementia in the UK Biobank

**DOI:** 10.3390/nu15010032

**Published:** 2022-12-21

**Authors:** Marilyn C. Cornelis, Puja Agarwal, Thomas M. Holland, Rob M. van Dam

**Affiliations:** 1 Department of Preventive Medicine, Northwestern University Feinberg School of Medicine, Chicago, IL 60611, USA; 2 Department of Internal Medicine, Rush University Medical Center, Chicago, IL 60612, USA; 3 Rush Alzheimer’s Disease Center, Rush University Medical Center, Chicago, IL 60612, USA; 4 Rush Institute for Healthy Aging, Rush University Medical Center, Chicago, IL 60612, USA; 5 Department of Exercise and Nutrition Sciences, Milken Institute School of Public Health, The George Washington University, Washington, DC 20052, USA

**Keywords:** diet pattern, dementia, cognition, genetics, interaction

## Abstract

A high adherence to the Mediterranean-Dietary Approaches to Stop Hypertension Diet Intervention for Neurodegenerative Delay (MIND) has been associated with better cognition and a lower risk of dementia in some but not all studies. We measured adherence to MIND and its association with cognitive health in the UK Biobank (UKB). A MIND score was derived from 24 h diet recall questionnaires for 120,661 participants who completed at least one of seven self-administered cognitive function tests. In a subset of 78,663 participants aged 55+, diagnosis of dementia was determined by linked hospital and death records. Multivariable regression and Cox proportional hazard ratio (HR) models were used to examine associations of MIND with cognitive ability and incident dementia. Higher adherence to MIND was associated with a small but significant worsening in performance on five of seven cognitive tests (*p* < 0.002). Associations were strongest among highly educated participants (*p* < 0.002 for MIND × education interaction). After a mean follow-up time of 10.5 years, 842 participants developed dementia. Overall, MIND adherence was not associated with incident dementia. An inverse association was observed among females (HR = 0.87 per score standard deviation (SD), *p* = 0.008) but not males (HR = 1.09, *p* = 0.11) (*p* = 0.008 for MIND × sex interaction). Similar associations with cognitive ability and dementia were observed for the Alternative Healthy Eating Index-2010 (AHEI-2010) dietary pattern. Associations were not modified by genetic susceptibility. In UKB, the MIND diet was not associated with better cognitive test scores and only with lower dementia risk in women.

## 1. Introduction

The prevalence of dementia is expected to increase due to progressive aging of the world population [1]. The Mediterranean-Dietary Approaches to Stop Hypertension Diet Intervention for Neurodegenerative Delay (MIND) is based on the most compelling evidence in the diet-dementia field and was initially characterized among US-based community older adults in 2015 [2,3]. MIND emphasizes consuming green leafy vegetables, berries, nuts, beans, whole grains, seafood, poultry, olive oil and wine and limiting intake of processed and red meat, fried and sugary foods, and high-saturated-fat foods [2,3]. Since 2015, MIND has been derived in other populations, often with modifications to account for diet assessment tools and differences in dietary behaviors. To our knowledge, seventeen population studies based in the US, Sweden, France, Australia, Spain, Netherlands, Brazil and Israel have investigated the relationship between MIND and cognitive health [2,4,5,6,7,8,9,10,11,12,13,14,15,16,17,18]. Five studies examined cognitive abilities [4,5,6,7,8]; four reported better cognitive performance with greater MIND adherence [4,5,6,7], while the fifth reported a cross-over interaction with income level [8]. Twelve studies examined cognitive decline [4,5,6,9,10,11,12,13,14,15,16,17]; seven reported slower declines in one or more cognitive abilities with greater MIND adherence [9,10,12,13,14,15], while another reported a similar pattern but only among a subset of older participants [11]. Four independent population studies examined incident dementia [2,16,18]; two reported a significantly lower risk of dementia with greater MIND adherence [2,16]; and a third also reported a lower risk of dementia, but the association waned with longer follow-up [18]. Importantly, MIND derivations in most studies were not fully described [4,6,7,10,11,12,13,15,17] or excluded diet components unique to this dietary pattern [10,12].

The current study estimated adherence to the MIND dietary pattern in the UK Biobank (UKB). Moreover, using this rich resource of genetic, phenotypic and follow-up data, we tested the hypothesis that high adherence to MIND is associated with better cognitive ability and a lower risk of incident dementia. We compared MIND findings with those of the Alternative Healthy Eating Index (AHEI)-2010 [19], another commonly used healthy dietary pattern score but not one specifically developed for protection against cognitive impairment.

## 2. Methods

### 2.1. Study Population

UKB is a population cohort of over 502,633 participants aged 37–73 years who underwent a comprehensive assessment at clinical centers across England, Wales and Scotland in 2006–2010 and whose health status is being tracked, in part, through linked death and health records [20]. The current analysis is based on participants who completed the Oxford WebQ, which was first administered in 2009–2010. This study was covered by the generic ethical approval for UK Biobank studies from the National Research Ethics Service Committee North West–Haydock (approval letter dated 17 June 2011, Ref 11/NW/0382), and all study procedures were performed in accordance with the World Medical Association Declaration of Helsinki ethical principles for medical research.

### 2.2. Diet Assessment (2009–2012)

Participants recruited in 2009–2010 completed the Oxford WebQ as part of the baseline assessment visit. Subsequently, all UKB participants with valid email addresses were invited to complete up to four WebQs at home, administered 3–4 months apart and on variable days of the week. The Oxford WebQ assesses up to 206 foods and 32 beverages over the previous 24 h. The quantity of each food/beverage item consumed was calculated by multiplying the assigned portion of each item by the amount consumed. Food compositions from the UK Nutrient Databank were used to calculate nutrient intakes [21]. For many food items, the size of a “serving” was not specified and, thus, were estimated based on how each question was asked, UK standard portion sizes and product information on packaging from different UK supermarkets [21]. The Oxford WebQ has been validated against biomarkers and interviewer-administered 24 h diet recalls [22,23]. WebQ also collected data on supplement use and the type of meals consumed. If a participant reported use of any supplement on the majority of WebQs, they were considered a regular supplement user. If a participant reported eating takeaway or restaurant meals on every WebQ, they were considered a regular fast meal consumer. The current analysis was limited to participants who completed at least two WebQs.

### 2.3. MIND Adherence Score

Mean intakes for each food item across completed WebQs were used to derive a MIND adherence score that closely aligned to that initially described for the Memory and Aging Population (MAP) [2,3]. MIND includes ten “brain healthy” components and five “brain unhealthy” components, as summarized in Table 1 for UKB (see Supplementary Table S1 for details). Each component corresponds to 1 point if adhered to, with a total max score of 15. Half points for partial adherence are also pre-specified. Noteworthy deviations from the original MIND score pertain to “olive oil”, “other vegetables” and “pastries and sweets” components. The questionnaire did not specifically ask about primary oil used for cooking, and thus, we assigned a point to participants reporting the use of olive oil in cooking every day if they reported using a fat/oil in cooking. Boiled/mashed white potatoes were not included in “other vegetables”. Sugar-sweetened beverages and sugar added to coffee, tea and cereal were included in “pastries and sweets”.

### 2.4. AHEI-2010 Adherence Score

We derived AHEI-2010 to serve as a negative control dietary pattern. AHEI-2010 was initially developed based on foods and nutrients predictive of chronic disease including cardiovascular disease, diabetes and cancer [19]. AHEI-2010 consists of 11 components: 6 emphasizing more vegetables, fruits, whole grains, nuts/legumes, omega-3 fatty acids and polyunsaturated fatty acids; 4 discouraging sugary beverages and fruit juices, red and processed meats, sodium and trans fatty acids; and a final component for moderate alcohol intake. Each component is scored on a scale of 0 (non-adherent) to 10 (fully adherent). The total score ranges from 0 to 110. Supplementary Table S2 provides the score derivation details for UKB. Omega-3 fatty acids were not estimated for UKB; thus, we applied a cutoff of 2 servings/week of “oily fish” for maximum score of 10 for that component per Chiuve et al. [19].

### 2.5. Cognitive Assessments

Self-administered computerized cognitive function tests were developed for UKB to enable population-scale cognitive testing and have been described in detail previously [24,25]. Touchscreen tests administered at the assessment centers (2006–2010) included prospective memory (PM), fluid intelligence (FI, verbal-numerical reasoning), pairs matching (Pairs, visual memory) and reaction time (RT) tests. In 2014, participants were invited to complete on-line tests at home, including the symbol digit substitution test (SDS, complex processing speed) and two Trail Making tests (Trail A/Trail B, visual search, scanning, processing speed, mental flexibility, and executive functions). Brief descriptions of each test are provided in the Supplementary Methods.

### 2.6. Dementia Outcomes

A new clinical diagnosis of all-cause dementia was derived by UKB a priori with hospital admission and death record data utilizing International Classification of Diseases version 10 (ICD-10 codes) [26]. When possible, all-cause dementia was further specified as Alzheimer’s, vascular or frontotemporal dementia. Prevalent cases were defined as those whose date of diagnosis was before or at the time of their first Oxford WebQ or who self-reported dementia at the baseline assessment visit (2006–2010).

### 2.7. Genetic Data and Calculation of Genetic Susceptibility Scores (GS)

All UKB participants were genotyped using genome-wide arrays. Quality control and imputation to the Haplotype Reference Consortium v1.1 and UK10K reference panels was performed centrally by the Wellcome Trust Centre for Human Genetics [27]. *APOE* carriers(ε4+) and non-carriers(ε4−) were defined using directly genotyped SNPs rs429358 and rs7412. A genetic susceptibility score (GS) for Alzheimer’s dementia (AD) was computed as described previously [16] using 25 SNPs (excluding *APOE* SNPs) with MAF > 1% and reaching significance level (*p* < 5 × 10^−8^) in genome-wide association studies of AD [28,29]. GS_AD_ uses the sum of the products of SNP risk alleles and their corresponding weights as GS_AD_ = Σ*_i_*^n^ log(OR*_ij_*) × G*_ij_* for the *i*th individual, where log(OR*_ij_*) = the log of the OR for the *j*th SNP, G*_ij_* = the number of risk alleles (0,1, or 2) for the *j*th SNP and *n* = 25 candidate SNPs. The score is rescaled according to the number of risk alleles (#SNPs × 2) to facilitate interpretation. A higher GS_AD_ corresponds to an elevated AD risk. We limited genetic analysis to unrelated individuals of British-European (EUR, ~96% of sample) genetically inferred ancestry based on a principal component analysis (PCA) by Pan-UKB [30]. Other ancestral groups presented with too few dementia cases to yield valid statistical tests.

### 2.8. Other Covariates

Prevalent depression, diabetes, hypertension, cardiovascular disease and stroke were defined using self-reported diagnoses, medication use and hospital admission data. Other covariate or confounder information was collected via self-report using the touchscreen or physical assessment as described in detail previously [20] and included sex, race/ethnicity, the Townsend deprivation index, education level, income level, employment status, smoking status, physical activity, Body Mass Index (BMI), self-rated health and family history of dementia. 

### 2.9. Statistical Analysis

All statistical analyses were performed using the SAS v9.4 statistical package (SAS Institute, Cary, NC, USA). Of the 120,790 consenting participants completing at least two Oxford WebQs, we excluded 95 with no cognitive test data and 34 prevalent cases of dementia, leaving a maximal sample size of 120,661. Sample sizes for each cognition test varied and ranged from 56,632 (for FI) to 120,115 (for RT).

We examined the association between MIND adherence, expressed in tertiles, and cognitive test scores using linear or logistic (PM test only) regression adjusting for age, sex and race/ethnicity (model 1). In the multivariable regressions, we further adjusted for smoking (never, past, current: <10, 10 to 19, 20+ cigarettes/d), Townsend deprivation index (quartiles), education (college or university degree, A levels/A sociology levels or equivalent, O levels/general certificate of secondary education or equivalent, certificate of secondary education or equivalent, national vocational qualification or higher national diploma or higher national certificate equivalent, or other professional qualifications), income (5 levels), employment status (employed, retired, other), BMI, physical activity (quartiles of moderate/vigorous activity minutes/week), self-reported health (excellent, good, fair, poor), family history of dementia, history of hypertension, diabetes, heart disease, stroke and depression, fast meal consumption and total energy intake (model 2). Missing indicator variables were constructed for BMI (0.2% missing), self-reported health (0.2%), income (9%), Townsend (0.1%) and physical activity (1.7%) to maximize sample size. A third model additionally adjusted for *APOE* ε4 carrier status and 20 PCs (for population stratification) but was limited to the genetic data set described above (*n* = 90,363). To test for linear trends, MIND was entered into the models as a continuous term. In sensitivity analysis, we excluded participants with a history of diabetes, cardiovascular disease and stroke. The same statistical approach was applied to the analysis of AHEI-2010 adherence and cognition. We additionally estimated effect sizes per 1 SD of score for each diet pattern to facilitate cross-diet comparisons. Statistical significance was defined as *p* < 0.007 after applying a correction for seven cognition tests.

For incident dementia, we limited analysis to the 78,663 participants who were at least 55 years of age to avoid missing cases of clinical dementia that may occur among younger participants had follow-up been extended. There were too few cases of vascular or frontotemporal dementia, and thus, we limited clinical endpoints to all-cause and Alzheimer’s dementia. Cox proportional hazards regression models examined the association between MIND adherence, expressed in tertiles, and incident dementia. To test for linear trends, MIND was entered into the model as a continuous term. UKB participants were considered at risk for dementia from baseline (date of their first Oxford WebQ) and were followed up until the date of first dementia diagnosis, death, loss to follow-up or October 2021 (last date of all-cause dementia reported), whichever came first. The proportionality of hazards assumption was assessed using time-dependent explanatory variables and Schoenfeld residuals techniques [31] and was satisfied. Models were adjusted as described for cognitive ability regression models. However, we further adjusted model 2 and model 3 for baseline global cognitive function. The latter was derived by taking the mean value of standardized RT and Pairs tests. tests with the most non-missing data. The 1265 (1.6%) participants missing information on this variable of cognitive function were excluded in models 2 and 3. In sensitivity analysis, we (i) excluded incident dementia cases within 5 years of follow-up; (ii) excluded participants with a baseline history of diabetes, cardiovascular disease and stroke; and (iii) performed a competing risk analysis since death was a censoring event and plausibly competes with dementia onset. The same statistical approach was applied to the analysis of AHEI-2010 adherence and dementia. We additionally estimated risk per 1 SD of score for each diet pattern. Statistical significance was defined as *p* < 0.05.

We screened for effect modification (interaction) by sex, education, *APOE* ε4 carrier status and GS_AD_ by including in multivariable regressions the cross-product term of MIND (or AHEI) and the modifying variable. Significant interactions were defined as *p* < 0.002, after applying a correction for testing four effect modifiers and eight outcome measures. Post hoc stratified analysis used the same dietary pattern score cut-points to define tertiles for the full sample (cognitive ability outcomes) or the age 55+ sample (dementia outcomes).

## 3. Results

### 3.1. Baseline Characteristics 

The mean (SD) MIND adherence score was 6.14 (1.94) for full sample and 6.23 (1.92) for those at least 55 years of age at the time of their first WebQ (baseline). Details for the 15 MIND components are presented in Supplementary Table S3. RT, Pairs, FI and PM tests administered at the assessment center were completed on average (SD) 1.65 (1.25), 1.65 (1.25), 0.53 (0.69), and 0.53 (0.69) years *before* the WebQ, respectively. Cognition tests completed on-line at home (SDS, Trail A/B) were completed on average 3.95 (SD = 0.62) years *after* the WebQ. Sample characteristics collected via touchscreen were collected on average (SD) 1.67 (1.25) years before the first WebQ. Participants with higher adherence to MIND were more likely to be female; to be non-smokers; to have a college/university degree; to have a higher household income; to be more physically active; to have better self-reported health; and to be less likely to report a history of diabetes, heart disease, hypertension and stroke (Table 2). Participants highly adherent to a MIND diet were also more likely to supplement with vitamins and minerals and less likely to be fast meal consumers. The corresponding characteristics for the subset of participants at least 55 years of age are presented in Supplementary Table S4.

The mean (SD) AHEI-2010 adherence score was 56.3 (13.9) for the full sample and 57.3 (13.7) for those at least 55 years of age at the time of their first WebQ. Details for the 11 AHEI components are presented in Supplementary Table S5. Sample characteristics by AHEI adherence scores are presented in Table 2 and Supplementary Table S4. MIND and AHEI scores were moderately correlated (Spearman *r* = 0.67).

### 3.2. MIND and AHEI Adherence and Cognitive Ability

When adjusting for age, sex and race/ethnicity, higher adherence to MIND was significantly associated with better performance in FI and RT tests but poorer performance in the Pairs test (Table 3). When models were further adjusted for measures of socioeconomic status (SES), medical history and lifestyle, higher adherence to MIND was significantly associated with poor performance in the FI, Pairs, SDS, Trail A and Trail B tests. The results were similar when restricted to participants of European ancestry and further adjusting for *APOE* ε4 status (model 3, results are not shown). Excluding participants with a history of diabetes, cardiovascular disease and stroke also yielded similar results (data not shown). We systematically examined model covariates to gain insight to covariates, driving the marked change in direction of effect estimates. Education and/or income were the primary confounders, as shown in Supplementary Table S6. Similar associations between MIND and cognitive ability was observed among the subsample of participants at least 55 years of age (Supplementary Table S7). We observed a statistically significant MIND×sex interaction (*p* = 0.0001) for Trail B. Higher adherence to MIND was significantly associated with lower Trail B performance in both females and males, but the effect estimate among females (β = 0.007, *p* < 0.0001) was larger than among males (β = 0.005, *p* < 0.0001). A similar but nominally significant MIND × sex interaction (*p* = 0.02) for Trail A was also observed. We observed statistically significant MIND×education interactions (*p* < 0.002) for FI, SDS, Trail A, Trail B and PM (Supplementary Table S8). Regardless of whether “higher education” was defined as at least Level 4+ qualifications (~73% of sample) or College/University degree (~47% of sample), the inverse association between high MIND adherence and low cognition test performance was stronger and, often exclusive to, the higher education strata. Non-carriers of *APOE* ε4 performed better than ε4-carriers for the Pairs, Trail A, and Trail B tests (*p* < 0.002) but not for the remaining three tests (*p* > 0.05). GS_AD_ was not associated with cognitive function (*p* > 0.05 for all cognition tests). Genetic factors did not modify the associations between MIND and cognitive ability (*p* > 0.05 for all interactions).

When adjusting for age, sex and race/ethnicity, higher adherence to AHEI-2010 was significantly associated with poorer performance in the Pairs, SDS, Trail and PM tests (Table 4). After multivariable adjustment, higher AHEI-2010 adherence was significantly associated with lower performance on all tests. Excluding participants with a history of diabetes, cardiovascular disease and stroke yielded similar results (data not shown). Effect estimates were also sensitive to education or income adjustment as shown for MIND but to a lesser degree (data not shown). Similar associations between AHEI and cognitive ability was observed among the subsample of participants at least 55 years of age (Supplementary Table S9). We observed statistically significant AHEI×education interactions for Trail A and Trail B (*p* < 0.002 for all interactions);,whereby the inverse association between high AHEI adherence and poor cognition test performance was stronger in the higher education strata. Genetic factors did not modify the associations between AHEI and cognitive ability (*p* > 0.05 for all interactions).

To gain insight into the clinical relevance of our cognitive ability results, we examined the association between each cognitive test and incident dementia among those at least 55 years of age. Better performance on each cognitive test was associated with a significantly lower risk of dementia (*p* < 0.002). Effect estimates per unit change in test scores are presented in Supplementary Table S10.

### 3.3. MIND and AHEI-2010 Adherence and Incident Dementia 

After a mean (SD) follow-up time of 10.5 (1.8) years, 842 UKB participants developed clinical dementia and 351 of these had Alzheimer’s dementia. Mean (SD) age of onset was 74.6 (4.3) and 74.9 (4.1) years for all-cause and Alzheimer’s dementia, respectively. When adjusting for age, sex and race/ethnicity, a higher MIND or AHEI adherence was associated with a significantly lower risk of incident all-cause dementia but not Alzheimer’s dementia (Table 5). However, neither diet score was significantly associated with incident dementia in multivariable adjusted models, nor when restricting to participants of European ancestry and further adjusting for *APOE* ε4 status (model 3, data not shown). Excluding dementia cases diagnosed within 5 years of follow-up (*n* = 152), excluding participants with a baseline history of diabetes, cardiovascular disease and stroke (*n* = 6831), or accounting for the competing risk of death did not substantially change these results.

We observed a nominal MIND×sex interaction (*p* = 0.008) for incident all-cause dementia, whereby adherence to MIND was significantly associated with a lower risk of dementia among females (HR = 0.71 T3 vs. T1) but not males (HR = 1.16 T3 vs. T1) (Supplementary Table S11). A similar pattern of results was observed for AHEI-2010 (females: HR = 0.77 males: HR = 1.01, T3 vs. T1), but the AHEI×sex interaction was weaker and not significant (*p* = 0.07)*. APOE* and GS_AD_ were each associated with all-cause (*p* < 0.0001) and Alzheimer’s dementia (*p* < 0.0001) but did not modify the associations between MIND/AHEI and dementia (*p* > 0.05 for interaction). Education level did not modify the association between MIND/AHEI and dementia (*p* > 0.05 for interaction).

### 3.4. Individual Diet Pattern Components and Cognitive Health

Supplementary Table S12 summarizes our exploratory analysis of individual MIND components with measures of cognitive ability and incident dementia. Higher green leafy intake, higher whole grain intake, olive oil use, lower cheese intake, lower processed and red meat intake, and lower sweets/pastries intake were generally associated with worse cognitive function test scores. In contrast, higher poultry and moderate wine intake were generally associated with better cognitive function. With regard to incident all-cause dementia, higher “other vegetable” intake was associated with lower risk (component score HR (95% CI): 0.80 (0.65, 0.99), *p* = 0.04 for trend) while lower butter/margarine (HR (95% CI): 1.51 (1.24, 1.83), *p* < 0.0001) and lower fast fried food (HR (95% CI): 1.18 (1.00, 1.39), *p* = 0.05) intake were associated with higher risk. Corresponding results for AHEI components are presented in Supplementary Table S13. Higher intake of (total) vegetables, fruit and nuts/legumes was generally associated with worse cognitive function test scores while higher PUFA (% of energy), lower sodium and moderate alcohol intake were generally associated with better cognitive function. Higher vegetable and higher fatty fish intake were weakly associated with a lower risk of all-cause (HR (95% CI): 0.97 (0.95, 1.00), *p* = 0.04) and Alzheimer’s (HR (95% CI): 0.97 (0.95, 1.00), *p* = 0.04) dementia, respectively.

## 4. Discussion

We found limited evidence supporting the benefits of adherence to the MIND dietary pattern for cognitive health in the UKB. Specifically, higher adherence to MIND was unexpectedly associated with worse performance on the cognitive function tests. Adherence to MIND was not associated with incident dementia in the overall population, although an inverse association in women was observed. An analysis of AHEI-2010, another “healthy” diet pattern, with cognitive function and dementia incidence yielded similar results.

The unexpected association between MIND adherence and worse cognitive function in UKB was especially evident after adjusting for SES (i.e., education and income level). Furthermore, these associations between MIND adherence and cognitive function were stronger among highly educated participants than in those with less education. While not derived specifically for dementia, the AHEI-2010 score has also been linked to better cognitive ability and a slower rate in cognitive decline in some but not all studies [32]. AHEI-2010 includes several components similar to those of MIND. Indeed, the two dietary patterns were correlated in UKB and presented with similar patterns of associations with cognitive function. Higher adherence to MIND and AHEI-2010 was associated with poor performance on tests regardless of whether tests were administered at the assessment center (before diet data collection) or at home (after diet data collection). These cognitive tests are not standard to the field but are moderately to strongly correlated with well-validated tests for the same cognitive domain, suggesting that UKB tests have adequate validity [24,33]. These tests were also associated with incident dementia in UKB, as shown in the current study and by others [34]. Other UKB studies of specific foods and cognitive ability have also yielded unexpected findings. Lower vegetable and fruit and higher red meat, processed meat and refined grains were associated with higher cognitive ability [35,36]. Greater adherence to other “healthy” dietary patterns was also linked to lower cognitive ability in this population [36]. Unexpected findings may be specific to cognitive abilities, since the same foods and dietary patterns have been associated with risk of dementia, cardiovascular disease, metabolic disease and sleep quality in the UKB in the hypothesized direction [37,38].

We are aware of at least two studies of dietary patterns and cognitive ability that also reported interactions with SES but with directions opposite to ours. In the Brazilian Longitudinal Study of Adult Health, greater MIND adherence was associated with better executive function among participants with high income but worse executive function (and global cognition) among participants with low income [8]. In the Quebec Longitudinal Study on Nutrition and Successful Aging, adherence to a prudent pattern (high in vegetables, fruits, fish, poultry and lower-fat dairy products) was related to higher global cognition at recruitment only among participants with higher income or education [39]. Adherence to a Western pattern (higher meats, potatoes, processed foods and higher-fat dairy products) was associated with lower performance only among participants with lower education [39]. To our knowledge, there is no biological explanation for *impaired* cognitive function with greater MIND adherence. The only MIND components consistent with expectations were the associations of higher poultry and moderate wine intake with better cognitive function. While we considered a comprehensive set of confounders in our analysis, it is possible that the studied diet patterns correlate with other lifestyle, medical or SES factors linked to impaired cognitive ability not considered or measured in UKB. The paradoxical results and particular impact of SES on effect estimates might also be a result of participant selection leading to collider bias [40]: UKB participants were more likely to be older, to be female and to live in less socioeconomically deprived areas than nonparticipants [41]. They also presented with a more favorable risk factor profile compared to the general population [41,42]. When our *statistically significant* findings for MIND (and AHEI-2010) and cognitive function are placed in the context of the relationship between cognitive function and dementia risk, the effect sizes are very small. For example, a point-reduction in the 13-item FI test associates with ~9% higher risk of dementia. One SD increase in MIND is associated with only a 0.07-point lower FI score.

Importantly, the worse cognitive performance we observed for higher adherence to MIND (and AHEI-2010) in cross-sectional analyses did not extend to the prospective analysis of dementia risk. Although initially associated with a lower risk of dementia in minimally adjusted models, MIND and AHEI-2010 were not associated with risk of dementia in the overall study population and in males when accounting for additional confounders. In contrast, among females, a one SD increase in MIND (AHEI) was associated with ~13% (14%) lower risk of all-cause dementia. To our knowledge, sex-specific associations between MIND or AHEI-2010 and dementia have not been reported. A mechanism underlying our finding is unclear but others have reported sex-specific differences in associations between lifestyle, medical, and SES factors and dementia risk [43,44,45]. Whether the MIND diet has a more beneficial effect in females than males or sex-specific confounding is at play warrants further study.

Despite a few adaptations, our UKB MIND adherence score closely aligned with that originally described by Morris et al., which was based on evidence in the diet-dementia field from 2015 [2,3]. Several prior efforts in population-based studies were not fully described [4,6,7,10,11,12,13,15,17]; excluded key components [10,12] such as olive oil, nuts and butter/margarine; or used cutpoints based on the intake distributions of the sample [10]. Moreover, a key component of the MIND score targets “extra virgin olive oil”, but diet assessment in several cohort studies (including UKB) did not specify extra virgin olive oil and grouped olive oil with other vegetable oils [2]. When exploring the individual MIND components, “lower butter/margarine” and “lower fast fried food” as defined in UKB stood out as particular outliers in the score; both were associated with a higher risk of dementia. To our knowledge, butter and (stick) margarine were originally considered an “unhealthy” component of MIND on the basis of their saturated and trans-fat content [46]. Since the administration of the WebQ in UKB, the amount of trans-fat in UK margarine, and food supply more generally, has been very low and, thus, may not be relevant to this MIND component in UKB [47]. Indeed, many UKB participant scored well on the trans-fat component specific to AHEI-2010 and that was not associated with incident dementia. Conversely, margarine may be an important source of PUFA, another AHEI-specific component, associated with better cognitive ability in the UKB. Nevertheless, when we replaced the “lower butter/margarine” component with a “low butter” component, the significant increased risk of dementia persisted (data not shown), suggesting butter (or a correlated behavior) per se may be protective. Fast foods were not specifically itemized in the WebQ. The “fast fried food” component was ultimately limited to fried poultry, fish and potato products and, thus, may not have captured the intended component. Nuances in deriving MIND (and other predetermined dietary patterns) in different populations may contribute to inconsistencies in the diet-dementia literature, and thus, the generalizability of MIND (as initially defined) for dementia prevention in other populations remains inconclusive.

The large sample size and extensive diet, phenotype and genetic data are strengths of the current study. However, limitations, beyond those discussed above, should be acknowledged. The diet-cognitive function associations reported are based on cross-sectional analysis and, thus, may be subject to reverse causation. We combined data from hospital and death records, which have been shown to improve sensitivity and specificity of detecting clinical dementia but likely underestimated the number of incident dementia cases [26]. Diet was collected within a defined period that may not have adequately captured earlier diet behaviors and might have a greater impact on the disease process leading to dementia. Finally, UKB is not representative of the source population and, therefore, cannot be readily extrapolated to the general UK population.

## 5. Conclusions

In summary, we found limited evidence supporting the benefits of MIND adherence for cognitive health in a large UK population sample. Adherence to MIND may be associated with a lower risk of dementia in females. However, potential confounding in MIND–cognitive function associations and the role of different components of the MIND score requires further study.

## Figures and Tables

**Table 1:** MIND adherence score in the UK Biobank ^1^.

Diet Component	Example Oxford WebQ Items	Component Score		
		0	0.5	1
Green leafy vegetables	cabbage/kale, lettuce, spinach	≤2 servings/wk	2 < 6 servings/wk	6+ servings/wk
Other vegetables	green beans, broccoli, butternut squash, carrot, cauliflower, celery, cucumber, leek, mushroom, sweet pepper, sprouts, sweetcorn, sweet potato	<5 servings/wk	5 < 7 servings/wk	7+ servings/wk
Berries	berries	<1 serving/wk	1 serving/wk	2+ servings/wk
Nuts	nuts, peanuts, seeds, peanut butter	<0.5 serving/wk	0.5–4 servings/wk	>4 servings/wk
Olive oil	Participant used olive oil in cooking across all diet records that reported using fat/oil in cooking	No		Yes
Butter/margarine	butter/margarine on bread/crackers and potatoes	<1 tsp/d	1–2 tsp/d	>2 tsp/d
Cheese (not low fat)	hard cheese, soft cheese, cheese spread, cottage cheese, mozzarella, goat’s cheese	6+ servings/wk	1 < 6 servings/wk	<1 servings/wk
Whole grains	porridge, whole-wheat cereal/breads, wholemeal pasta, brown rice	<1 servings/d	1–2 servings/d	>2 servings/d
Fish (not fried)	tinned tuna, oily fish, white fish, prawns, lobster/crab, shellfish	<1 servings/m	1+ servings/m	1+ servings/wk
Beans	baked bean, pulses, broad bean, hummus, tofu	<1 serving/wk	1–3 servings/wk	>3 servings/wk
Poultry (not fried)	poultry	<1 serving/wk	1 serving/wk	2+ servings/wk
Red meat and products	sausage, beef, pork, lamb, bacon, ham	>6 servings/wk	4–6 servings/wk	<4 servings/wk
Fast/fried foods	fried poultry, breaded fish, fried potatoes, crisp/chips	>3 servings/wk	1–3 servings/wk	<1 serving/wk
Pastries and sweets	pastry, crumble, pancake, pudding, ice-cream, cake, doughnut, chocolate bar, biscuits, hot chocolate, fizzy drink, added sugars and preserves	7+ servings/wk	5–6 servings/wk	<5 servings/wk
Wine	red wine, rose wine, white wine, fortified wine	<1 serving/m	1 serving/m to 1 serving/wk	2–7 servings/wk

^1^ See Supplementary Table S1 for details.

**Table 2:** Baseline characteristics of participants by tertiles (T) of MIND and AHEI-2010 score ^1^.

Characteristic	MIND			AHEI-2010		
	T1 0.0–5.5 *n* = 40,256	T2 5.5–6.5 *n* = 35,963	T3 7.0–14.5 *n* = 44,442	T1 11.2–49.9 *n* = 40,220	T2 49.9–62.3 *n* = 40,221	T3 62.3–108.6 *n* = 40,220
Age, years	57.3 ± 8.0	57.9 ± 7.9	58.3 ± 7.7	56.9 ± 8.1	58.1 ± 7.8	58.6 ± 7.6
Female, *n* (%)	17,443 (43.3)	20,414 (56.8)	30,209 (68.0)	17,801 (44.3)	23,098 (57.4)	27,167 (67.6)
Race/ethnicity, *n* (%) White South Asian Black Chinese Other, Unknown	39,135 (97.2) 295 (0.7) 277 (0.7) 70 (0.2) 479 (1.2)	34,791 (96.7) 364 (1.0) 249 (0.7) 79 (0.2) 480 (1.3)	42,692 (96.0) 431 (1.0) 394 (0.9) 166 (0.4) 759 (1.7)	39,051 (97.1) 270 (0.7) 300 (0.8) 84 (0.2) 515 (1.3)	38,919 (96.8) 363 (0.9) 292 (0.7) 87 (0.2) 560 (1.4)	38,648 (96.1) 457 (1.1) 328 (0.8) 144 (0.4) 643 (1.6)
Townsend	−1.61 ± 2.85	−1.72 ± 2.80	−1.64 ± 2.84	−1.59 ± 2.86	−1.72 ± 2.80	−1.65 ± 2.84
Household income ₤52,000+, *n* (%)	11,009 (27.3)	10,895 (30.3)	14,695 (33.0)	12,451 (31.0)	12,072 (30.0)	12,076 (30.0)
College/university degree, *n* (%)	16,012 (39.8)	16,848 (46.9)	23,855 (53.7)	16,860 (41.9)	18,781 (46.7)	21,073 (52.4)
Currently employed, *n* (%)	25,171 (62.5)	21,692 (60.3)	26,433 (59.5)	25,657 (63.8)	24,066 (59.8)	23,573 (58.6)
Current smoker, *n* (%)	3898 (9.7)	2348 (6.5)	2237 (5.0)	4097 (10.2)	2628 (6.5)	1758 (4.4)
BMI, kg/m^2^	27.4 ± 4.8	26.7 ± 4.5	26.0 ± 4.3	27.6 ± 4.8	26.7 ± 4.5	25.8 ± 4.3
Moderate to vigorous physical activity, hours/week	68.7 ± 86.0	70.8 ± 78.9	75.2 ± 79.1	67.3 ± 82.7	71.3 ± 80.5	76.6 ± 80.9
Self-reported health, *n* (%) Excellent Good Fair Poor	7172 (17.8) 23,733 (59.1) 7824 (19.5) 1456 (3.6)	7950 (22.1) 21,536 (60.0) 5562 (15.5) 848 (2.4)	11,015 (24.8) 26,856 (60.5) 5680 (12.8) 812 (1.8)	7386 (18.4) 23,617 (58.8) 7710 (19.2) 1441 (3.6)	8725 (21.7) 24,228 (60.4) 6231 (15.5) 964 (2.4)	10,026 (25.0) 24,280 (60.5) 5125 (12.8) 711 (1.8)
Family history of dementia, *n* (%)	829 (2.1)	803 (2.2)	1107 (2.5)	821 (2.0)	941 (2.3)	977 (2.4)
Diabetes, *n* (%)	1339 (3.3)	898 (2.5)	841 (1.9)	1167 (2.9)	1049 (2.6)	862 (2.1)
Heart disease, *n* (%)	1820 (4.5)	1362 (3.8)	1409 (3.2)	1725 (4.3)	1533 (3.8)	1333 (3.3)
Hypertension, *n* (%)	7329 (18.2)	5955 (16.6)	6592 (14.8)	7340 (18.3)	6585 (16.4)	5951 (14.8)
Stroke, *n* (%)	494 (1.2)	392 (1.1)	393 (0.9)	498 (1.2)	414 (1.0)	367 (0.9)
Depression, *n* (%)	1836 (4.6)	1444 (4.0)	1772 (4.0)	1752 (4.4)	1695 (4.2)	1605 (4.0)
*APOE* ε4 carriers, *n* (%) ^2^	9403 (27.7)	8578 (28.1)	10,600 (28.0)	9344 (27.5)	9416 (27.6)	9821 (28.7)
Genetic Score (GS)_AD_^2^	26.9 ± 3.0	26.9 ± 3.1	26.8 ± 3.1	26.9 ± 3.1	26.9 ± 3.1	26.9 ± 3.1
Energy, calories/d	2075 ± 440	2009 ± 428	1953 ± 425	2097 ± 440	1990 ± 430	1945 ± 418
Frequent fast meal consumer, %	1483 (3.7)	943 (2.6)	835 (1.9)	1601 (4.0)	1024 (2.6)	636 (1.6)
Vitamin/mineral supplement user, %	16,639 (41.3)	16,938 (47.1)	23,759 (53.5)	16,545 (41.1)	19,030 (47.3)	21,761 (54.1)

^1^ Values are mean ± SD or *n* (%). Age at the time of first Oxford WebQ. Non-diet data collected at baseline assessment center visit or linked hospital records. ^2^ *n* = 33,944, 30,546, 37,857 for MIND T1, T2 and T3, respectively. *n* = 33,987, 34,131, 34,229 for AHEI T1, T2 and T3, respectively.

**Table 3:** Associations between adherence to MIND and cognitive ability.

Score Tertile	*n*	Test Score ^5^	Model 1 ^1^		Model 2 ^2^	
			β (95% CI)	*p*	β (95% CI)	*p*
^3^ Fluid Intelligence (higher scores reflect better performance)						
MIND T1	18,764	6.69 (2.07)	Reference		Reference	
MIND T2	16,865	6.73 (2.05)	0.11 (0.07, 0.15)	<0.0001	−0.03 (−0.07, 0.007)	0.12
MIND T3	21,003	6.69 (2.02)	0.12 (0.08, 0.16)	<0.0001	−0.14 (−0.18, −0.10)	<0.0001
MIND score (raw), linear trend MIND score (per SD), linear trend			0.03 (0.02, 0.03) 0.05 (0.03, 0.07)	<0.0001	−0.04 (−0.05, −0.03) −0.07 (−0.09, −0.06)	<0.0001
^4^ Reaction Time (lower scores reflect better performance)						
MIND T1	40,084	542 (101)	Reference		Reference	
MIND T2	35,807	544 (99)	−2.35 (−3.72, −0.97)	0.0008	−0.47 (−1.85, 0.91)	0.51
MIND T3	44,224	548 (101)	−2.52 (−3.85, −1.19)	0.0002	0.66 (−0.70, 2.02)	0.34
MIND score (raw), linear trend MIND score (per SD), linear trend			−0.45 (−0.74, −0.16) −0.87 (−1.43, −0.32)	0.002	0.33 (0.03, 0.62) 0.63 (0.06, 1.21)	0.03
^4^ Pairs Matching (lower scores reflect better performance)						
MIND T1	39,941	1.38 (0.62)	Reference		Reference	
MIND T2	35,609	1.39 (0.62)	0.008 (−0.001, 0.02)	0.09	0.01 (0.001, 0.02)	0.03
MIND T3	43,999	1.42 (0.62)	0.03 (0.02, 0.03)	<0.0001	0.03 (0.02, 0.04)	<0.0001
MIND score (raw), linear trend MIND score (per SD), linear trend			0.007 (0.005, 0.009) 0.01 (0.01, 0.2)	<0.0001	0.008 (0.006, 0.01) 0.02 (0.01, 0.02)	<0.0001
^3^ Symbol Digit Substitution (higher scores reflect better performance)						
MIND T1	23,075	20.2 (5.1)	Reference		Reference	
MIND T2	21,153	20.1 (5.1)	0.11 (0.03, 0.20)	0.01	−0.07 (−0.15, 0.02)	0.16
MIND T3	26,404	19.9 (5.0)	0.06 (−0.02, 0.15)	0.13	−0.25 (−0.33, −0.16)	<0.0001
MIND score (raw), linear trend MIND score (per SD), linear trend			0.004 (−0.01, 0.02) 0.007 (−0.03, 0.04)	0.68	−0.07 (−0.09, −0.05) −0.14 (−0.17, −0.10)	<0.0001
^4^ Trail A (lower scores reflect better performance)						
MIND T1	20,603	3.58 (0.32)	Reference		Reference	
MIND T2	18,780	3.59 (0.32)	−0.003 (−0.009, 0.003)	0.34	0.005 (−0.001, 0.01)	0.10
MIND T3	23,258	3.60 (0.31)	−0.002 (−0.007, 0.004)	0.56	0.01 (0.007, 0.02)	<0.0001
MIND score (raw), linear trend MIND score (per SD), linear trend			−0.0007 (−0.002, 0.0006) −0.001 (−0.004, 0.001)	0.31	0.003 (0.002, 0.004) 0.006 (0.003, 0.008)	<0.0001
^4^ Trail B (lower scores reflect better performance)						
MIND T1	20,603	4.10 (0.33)	Reference		Reference	
MIND T2	18,780	4.12 (0.33)	−0.003 (−0.009, 0.003)	0.35	0.01 (0.005, 0.02)	0.0002
MIND T3	23,257	4.13 (0.33)	−0.001 (−0.007, 0.004)	0.62	0.02 (0.02, 0.03)	<0.0001
MIND score (raw), linear trend MIND score (per SD), linear trend			0.0001 (−0.001, 0.001) 0.0002 (−0.002, 0.003)	0.87	0.006 (0.005, 0.008) 0.012 (0.010, 0.015)	<0.0001
^4^ Prospective Memory Test (higher scores reflect better performance)						
	*n*	% correct	OR (95% CI)	*p*	OR (95% CI)	*p*
MIND T1	18,887	86	Reference		Reference	
MIND T2	16,978	86	1.05 (0.99, 1.12)	0.11	1.01 (0.95, 1.07)	0.78
MIND T3	21,132	85	1.03 (0.97, 1.09)	0.30	0.95 (0.90, 1.01)	0.11
MIND score (raw), linear trend MIND score (per SD), linear trend			1.00 (0.99, 1.01) 1.00 (0.98, 1.03)	0.96	0.98 (0.97, 0.99) 0.96 (0.94, 0.99)	0.004

^1^ Model 1: adjusted for age, sex and self-reported race/ethnicity. ^2^ Model 2: adjusted for age, sex, race/ethnicity, education, Townsend deprivation index, income, employment status, family history of dementia; history of hypertension, diabetes, heart disease, stroke and depression; self-reported health, smoking, physical activity, BMI, fast meal consumption and energy intake. ^3^ Positive beta-coefficients for FI (difference in 13-point score) and SDS (difference in number of correct substitutions) and OR > 1 for PM (correct on first attempt) correspond to higher performance compared to MIND tertile 1. ^4^ Negative beta-coefficients for Pairs (difference in (log-transformed) number of errors), RT (difference in time, milliseconds, to respond), Trails A and Trails B (difference in (log transformed) time, deciseconds, to complete) correspond to higher performance compared to MIND tertile 1. ^5^ Data are raw mean (SD) scores of each cognitive function test.

**Table 4:** Associations between adherence to AHEI-2010 and cognitive ability.

Score Tertile	*n*	Test Score ^3^	Model 1 ^1^		Model 2 ^2^	
			β (95% CI)	*p*	β (95% CI)	*p*
^4^ Fluid Intelligence (higher scores reflect better performance)						
AHEI T1	18,826	6.77 (2.06)	Reference		Reference	
AHEI T2	18,819	6.72 (2.06)	0.02 (−0.02, 0.07)	0.25	−0.05 (−0.09, −0.008)	0.02
AHEI T3	18,987	6.63 (2.02)	−0.01 (−0.05, 0.03)	0.60	−0.17 (−0.21, −0.13)	<0.0001
AHEI score (raw), linear trend AHEI score (per SD), linear trend			−0.0002 (−0.001, 0.001) −0.003 (−0.02, 0.01)	0.72	−0.006 (−0.007, −0.004) −0.08 (−0.09, −0.06)	<0.0001
^5^ Reaction Time (lower scores reflect better performance)						
AHEI T1	40,056	538 (99)	Reference		Reference	
AHEI T2	40,033	546 (100)	−0.01 (−1.35, 1.33)	0.99	1.23 (−0.12, 2.57)	0.07
AHEI T3	40,025	551 (102)	0.65 (−0.71, 2.01)	0.35	2.77 (1.37, 4.16)	<0.0001
AHEI score (raw), linear trend AHEI score (per SD), linear trend			0.03 (−0.01, 0.07) 0.42 (−0.14, 0.98)	0.14	0.10 (0.06, 0.14) 1.41 (0.83, 1.99)	<0.0001
^5^ Pairs Matching (lower scores reflect better performance)						
AHEI T1	39,919	1.36 (0.62)	Reference		Reference	
AHEI T2	39,852	1.40 (0.62)	0.03 (0.02, 0.04)	<0.0001	0.03 (0.02, 0.04)	<0.0001
AHEI T3	39,776	1.43 (0.63)	0.04 (0.03, 0.05)	<0.0001	0.04 (0.03, 0.05)	<0.0001
AHEI score (raw), linear trend AHEI score (per SD), linear trend			0.001 (0.0012, 0.002) 0.02 (0.016, 0.023)	<0.0001	0.001 (0.0011, 0.0017) 0.019 (0.016, 0.023)	<0.0001
^4^ Symbol Digit Substitution (higher scores reflect better performance)						
AHEI T1	23,018	20.4 (5.1)	Reference		Reference	
AHEI T2	23,585	20.0 (5.1)	−0.06 (−0.14, 0.03)	0.18	−0.19 (−0.27, −0.11)	<0.0001
AHEI T3	24,029	19.7 (5.1)	−0.19 (−0.27, −0.10)	<0.0001	−0.40 (−0.49, −0.32)	<0.0001
AHEI score (raw), linear trend AHEI score (per SD), linear trend			−0.006 (−0.009, −0.004) −0.08 (−0.12, −0.05)	<0.0001	−0.013 (−0.016, −0.011) −0.18 (−0.22, −0.15)	<0.0001
^5^ Trail A (lower scores reflect better performance)						
AHEI T1	20,614	3.57 (0.32)	Reference		Reference	
AHEI T2	20,829	3.59 (0.32)	0.003 (−0.003, 0.009)	0.29	0.009 (0.003, 0.01)	0.002
AHEI T3	21,198	3.61 (0.31)	0.01 (0.005, 0.02)	0.0005	0.02 (0.01, 0.03)	<0.0001
AHEI score (raw), linear trend AHEI score (per SD), linear trend			0.0003 (0.0001, 0.0005) 0.004 (0.002, 0.006)	0.001	0.0006 (0.0004, 0.0008) 0.008 (0.006, 0.01)	<0.0001
^5^ Trail B (lower scores reflect better performance)						
AHEI T1	20,614	4.09 (0.33)	Reference		Reference	
AHEI T2	20,828	4.12 (0.33)	0.004 (−0.001, 0.01)	0.14	0.015 (0.009, 0.021)	<0.0001
AHEI T3	21,198	4.14 (0.33)	0.016 (0.01, 0.021)	<0.0001	0.034 (0.028, 0.039)	<0.0001
AHEI score (raw), linear trend AHEI score (per SD), linear trend			0.0005 (0.0003, 0.0007) 0.007 (0.004, 0.009)	<0.0001	0.0011 (0.0009, 0.0012) 0.015 (0.012, 0.017)	<0.0001
^4^ Prospective Memory Test (higher scores reflect better performance)						
	*n*	% correct	OR (95% CI)	*p*	OR (95% CI)	*p*
AHEI T1	18,927	87	Reference		Reference	
AHEI T2	18,958	85	0.91 (0.86, 0.97)	0.002	0.89 (0.84, 0.95)	0.0003
AHEI T3	19,098	85	0.94 (0.88, 1.00)	0.04	0.90 (0.85, 0.96)	0.002
AHEI score (raw), linear trend AHEI score (per SD), linear trend			1.00 (0.99, 1.00) 0.96 (0.94, 0.99)	0.003	1.00 (0.99, 1.00) 0.95 (0.92, 0.97)	<0.0001

^1^ Model 1: adjusted for age, sex and self-reported race/ethnicity. ^2^ Model 2: adjusted for age, sex, race/ethnicity, education, Townsend deprivation index, income, employment status, family history of dementia; history of hypertension, diabetes, heart disease, stroke and depression; self-reported health, smoking, physical activity, BMI, fast meal consumption and energy intake. ^3^ Data are raw mean (SD) scores of each cognitive function test. ^4^ Positive beta-coefficients for FI (difference in 13-point score) and SDS (difference in number of correct substitutions) and OR > 1 for PM (correct on first attempt) correspond to higher performance compared to AHEI tertile 1. ^5^ Negative beta-coefficients for Pairs (difference in (log-transformed) number of errors), RT (difference in time, milliseconds, to respond), Trails A and Trails B (difference in (log transformed) time, deciseconds, to complete) correspond to higher performance compared to AHEI tertile 1.

**Table 5:** MIND/AHEI-2010 adherence and incident dementia (age 55+ years at baseline).

Model	Cases/ Person-Years	MIND Score				Cases/ Person-Years	AHEI Score			
		Model 1 ^1^		Model 2 ^2^			Model 1 ^1^		Model 2 ^2^	
		HR (95% CI)	*p*	HR (95% CI)	*p*		HR (95% CI)	*p*	HR (95% CI)	*p*
All-Cause Dementia										
T1	283/259,772	Ref		Ref		298/273,476	Ref		Ref	
T2	348/324,304	0.98 (0.84, 1.15)	0.79	1.06 (0.90, 1.24)	0.51	282/276,915	0.90 (0.77, 1.07)	0.23	0.93 (0.78, 1.10)	0.38
T3	211/245,160	0.81 (0.68, 0.97)	0.03	0.90 (0.74, 1.09)	0.27	262/278,666	0.84 (0.71, 1.00)	0.04	0.89 (0.75, 1.06)	0.20
Diet score (raw), linear trend Diet score (per SD), linear trend	842/829,236	0.96 (0.93, 1.00) 0.93 (0.86, 1.00)	0.04	0.99 (0.95, 1.03) 0.97 (0.90, 1.05)	0.48	842/829,236	0.99 (0.99, 1.00) 0.92 (0.85, 0.98)	0.01	1.00 (0.99, 1.00) 0.94 (0.87, 1.01)	0.10
Alzheimer’s Dementia										
T1	117/259,807	Ref		Ref		117/273,683	Ref		Ref	
T2	139/324,333	0.94 (0.74, 1.21)	0.63	1.00 (0.78, 1.30)	0.98	116/276,938	0.94 (0.73, 1.22)	0.65	0.94 (0.72, 1.23)	0.66
T3	95/245,184	0.88 (0.67, 1.16)	0.35	0.96 (0.72, 1.28)	0.76	118/278,703	0.96 (0.74, 1.24)	0.75	0.99 (0.75, 1.29)	0.91
Diet score (raw), linear trend Diet score (per SD), linear trend	351/829,324	0.99 (0.93, 1.05) 0.98 (0.88, 1.09)	0.67	1.01 (0.95, 1.07) 1.02 (0.91, 1.14)	0.76	351/829,324	1.00 (0.99, 1.01) 1.00 (0.90, 1.12)	0.97	1.00 (0.99, 1.01) 1.01 (0.90, 1.14)	0.82

^1^ Model 1: Results from Cox-proportional hazard models adjusted for age, sex and self-reported race/ethnicity (*n* = 78,663). ^2^ Model 2: Results from Cox-proportional hazard models adjusted for age, sex, self-reported race/ethnicity, education, Townsend deprivation index, income, employment status, global cognition score, family history of dementia; history of hypertension, diabetes, heart disease, stroke and depression; self-reported health, smoking, physical activity, BMI, fast meal consumption and energy intake (*n* = 77,398).

## Data Availability

All data for this manuscript can be obtained upon approval from the UK Biobank at https://www.ukbiobank.ac.uk/enable-your-research/apply-for-access (accessed on 1 January 2021).

## Supplementary Materials

The following supporting information can be downloaded at: https://www.mdpi.com/article/10.3390/nu15010032/s1. Supplementary Methods: Cognitive function tests; Supplementary Table S1: MIND score derivation in UK Biobank; Supplementary Table S2: AHEI-2010 score derivation in UK Biobank; Supplementary Table S3: MIND components in UK Biobank; Supplementary Table S4: Baseline characteristics of participants 55+ years of age by tertiles of MIND and AHEI-2010 score; Supplementary Table S5: AHEI-2010 components in UK Biobank; Supplementary Table S6: Associations between adherence to MIND and cognitive ability with and without adjustment for education and income level; Supplementary Table S7: Associations between adherence to MIND and cognitive ability among participants aged 55+ years; Supplementary Table S8: MIND score×education (higher vs. lower) interaction tests; Supplementary Table S9: Associations between adherence to AHEI and cognitive ability among participants aged 55+ years; Supplementary Table S10: Cognitive ability and incident dementia; Supplementary Table S11: MIND/AHEI-2010 adherence and incident dementia by sex; Supplementary Table S12: Individual MIND components and cognitive health; Supplementary Table S13: Individual AHEI-2010 components and cognitive health; Supplementary References.
