# Supplementary material for: MIND Dietary Pattern and Its Association with Cognition and Incident Dementia in the UK Biobank
Source: Nutrients. 2022 Dec 21;15(1):32. doi: 10.3390/nu15010032 (PMC9823700; doi:10.3390/nu15010032)
Supplement: Supplementary file 1 [file nutrients-15-00032-s001.zip › nutrients-2085494-supplementary.pdf]

Supplementary Materials

Supplementary Methods

Cognitive function

Tests administered at the assessment center (2006–2010) and in the following order include: Prospective memory (Part 1), Pairs matching, Fluid intelligence, Reaction time and Prospective memory (Part 2). Beginning in 2014, the following tests were completed on−line and at home: Fluid intelligence (excluded from current analysis), Trail Making Test A, Trail Making Test B, Symbol Digit substitution and then Pairs matching (excluded from current analysis). A digit recall test was only performed in a subset of the participants at baseline and was phased out during the latter part of recruitment and continued on−line. This test was not included for analysis given the different environments in which the tests were completed.

Prospective Memory (PM) Test: This test was added part−way through the baseline assessment period and completed at the assessment centers. Participants were given the following instructions: 'At the end of the games we will show you four colored symbols and ask you to touch the blue square. However, to test your memory, we want you to actually touch the Orange Circle instead'. Participants were scored as zero or one, depending on whether they completed the task on first attempt or not. This test assesses PM: the ability to carry out future intentions at a specific time or in response to a specific event and therefore includes a prospective component (remembering to remember) and a retrospective component (remembering the content of what is to be remembered) [1,2].

Pairs Matching (Pairs) Test: This episodic visual memory test was completed at the assessment centers. Participants were shown 6 pairs of cards for 5 seconds, which were then turned over. Participants were instructed to select, from recall and in the fewest number of attempts, the pairs of cards that had matching symbols. There was no time limit and the participants could make as many attempts as they needed to find all the pairs. The memory test score in the present study is the total number of errors made during this task until the six pairs of identical cards were touched consecutively. We restricted our analyses to individuals who finished the test and log (+1) transformed the number of errors for the analysis. Transformation was necessary since the distribution of raw scores were zero−inflated and highly skewed to the right. During the pilot phase of the UK Biobank, a subset of participants completed this test twice in immediate succession and the intraclass correlation was 0.17 [3].

Fluid Intelligence (FI) Test: This test was added part−way through the baseline assessment period and completed at the assessment centers. Participants were presented with 13 verbal logic/reasoning−type multiple choice questions and had to answer as many as they could within 2 minutes. There were six verbal items and seven numerical items, involving sequence recognition and arithmetic. Incorrect or unattempted questions were scored as zero. The total number of correct answers (max 13) was normally distributed and used for our analysis. The Cronbach alpha coefficient for these items has been reported elsewhere as 0.62 [4]. The UK Biobank describes FI as the "capacity to solve problems that require logic and

reasoning ability, independent of acquired knowledge" [5]. Others have preferred the test label 'verbal−numerical reasoning' as opposed to the UK Biobank's label 'fluid Intelligence" since performance on some items more likely rely on crystalized knowledge" [3,4,6−12].

Reaction Time (RT) Test: For this measure of simple processing speed, participants completed a timed test of symbol matching at the assessment centers. Participants were shown one pair of cards out of a set of 12 pairs. If both cards displayed a matching symbol, participants pressed a response button as quickly as possible using their dominant hand. Symbols included: equals−sign "=", 3 overlapping triangles, hollow circle, hollow square, ruby−post "H", smiley face, solid circle, solid square, triangle and cross "+". Five 'training' trials were administered, followed by seven test trials. The score for analysis was the mean time (in milliseconds) to press the button, derived from the four trials in which a matching pair occurred. The UK Biobank indicates that i) times under 50 ms must be due to anticipation rather than reaction and so were excluded and ii) times over 2000 ms are ignored since cards had disappeared by then. For the current analysis additional potential outliers were truncated to 4 SD away from the mean. This approach generated better normally distributed scores than log−transformed scores. However, results were similar when log−transformed values were analyzed (data not shown). Cronbach's alpha for this task has previously been reported as 0.85 [4].

Symbol Digit Substitution (SDS) Test: This test for complex processing speed was completed at follow−up (~2014) on home computers and involves matching numbers to a set of symbols. Participants are presented with symbols paired with digits and are asked to enter the digits that are paired with the symbols in the empty spaces. We used the number of correct substitutions for our analyses. Potential outliers were truncated to 4 SD away from the mean.

Trail Making Test A and B: These visual attention tests (or executive function) provide information on visual search, scanning, speed of processing, mental flexibility, and executive functions and were completed at follow−up on home computers (~2014). Participants were asked to connect scattered circles containing a sequence of numbers (Trail A) and then to connect circles containing numbers or letters by alternating between them in ascending sequence (Trail B). We used the time taken to complete these tests for our analyses, and these data were log−transformed to account for the right−skewed distribution of the raw scores.

Supplementary Table S1. MIND score derivation in UK Biobank.

Diet component	UKB variable#	Label	Coding (standard servings per day)	Component Score		
				0	0.5	1
Green leafy vegetables	103990	Veg consumer (dep)	0/1, N/Y			
	104090	Side salad (1/2)	444/555/1/2/300, ¼ / ½ /1/2/3+			
	104160	Cabbage/kale	444/555/1/2/300, ¼ / ½ /1/2/3+			
	104240	Lettuce	444/555/1/2/300, ¼ / ½ /1/2/3+			
	104300	Spinach	444/555/1/2/300, ¼ / ½ /1/2/3+			
	104370	Watercress	444/555/1/2/300, ¼ / ½ /1/2/3+			
	104080	Coleslaw (cabbage)	444/555/1/2/300, ¼ / ½ /1/2/3+			
		*1 serving side salad = ½ serving greens + ½ serving "other vegetable"				
	Used mean intake across diet records for each item then summed item means for daily servings. Applied points.			≤2/wk	2<6/wk	6+/wk
Other vegetables	103990	Veg consumer (dep)	0/1, N/Y			
	104090	Side salad (1/2)	444/555/1/2/300, ¼ / ½ /1/2/3+			
	104060	Mixed veg	444/555/1/2/300, ¼ / ½ /1/2/3+			
	104070	Veg pieces	444/555/1/2/300, ¼ / ½ /1/2/3+			
	104120	Green beans	444/555/1/2/300, ¼ / ½ /1/2/3+			
	104130	Beet root	444/555/1/2/300, ¼ / ½ /1/2/3+			
	104140	Broccoli	444/555/1/2/300, ¼ / ½ /1/2/3+			
	104150	Butternut squash	444/555/1/2/300, ¼ / ½ /1/2/3+			
	104170	Carrot	444/555/1/2/300, ¼ / ½ /1/2/3+			
	104180	Cauliflower	444/555/1/2/300, ¼ / ½ /1/2/3+			
	104190	Celery	444/555/1/2/300, ¼ / ½ /1/2/3+			
	104200	Courgetti/zuch	444/555/1/2/300, ¼ / ½ /1/2/3+			
	104210	Cucumber	444/555/1/2/300, ¼ / ½ /1/2/3+			
	104220	Garlic	444/555/1/2/300, ¼ / ½ /1/2/3+			
	104230	Leek	444/555/1/2/300, ¼ / ½ /1/2/3+			
	104250	Mushroom	444/555/1/2/300, ¼ / ½ /1/2/3+			
	104260	Onion	444/555/1/2/300, ¼ / ½ /1/2/3+			
	104270	Parsnip	444/555/1/2/300, ¼ / ½ /1/2/3+			
	104280	Pea (frozen, fresh, tinned)	444/555/1/2/300, ¼ / ½ /1/2/3+			
	104290	Sweet pepper	444/555/1/2/300, ¼ / ½ /1/2/3+			
	104310	Sprouts	444/555/1/2/300, ¼ / ½ /1/2/3+			

	104320	Sweetcorn	444/555/1/2/300, ¼ / ½ /1/2/3+			
	104330	Sweet potato	444/555/1/2/300, ¼ / ½ /1/2/3+			
	104340	Fresh tomato	444/555/1/2/300, ¼ / ½ /1/2/3+			
	104350	Tinned tomato	444/555/1/2/300, ¼ / ½ /1/2/3+			
	104360	Turnip/swede	444/555/1/2/300, ¼ / ½ /1/2/3+			
	104380	Other veg	444/555/1/2/300, ¼ / ½ /1/2/3+			
	102520	Soup consumer (dep)	0/1, N/Y			
	102540	Canned (dep)	555/1/2/3/400, ½ /1/2/3/4+			
	102620	Homemade (dep)	555/1/2/3/400, ½ /1/2/3/4+			
	20108	Ingredients in canned soup	5, vegetables			
	20109	Ingredients in homemade soup	5, vegetables			
	103310	Spreads/sauces consumers (dep)	0/1, N/Y			
	20088	Types of spreads/sauces consumed				
		Tomato sauce	346 (max 1 serving)			
		*unable to separate pure fruit/veg juice<br>*1veg soup= ¼ veg serving				
	Used mean intake across diet records for each item then summed item means for daily servings. Applied points			<5/wk	5<7/wk	7+/wk
Berries	104400	Fruit consumer (dep)	0/1, N/Y			
	104470	Berry	555/1/2/3/400, ½ /1/2/3/4+			
	Used mean intake across diet records for daily servings. Applied points			<1/wk	1/wk	2+/wk
Nuts	102400	Savory snack consumer (dep)	0/1, N/Y			
	102430	Salted nuts	555/1/2/300, ½ /1/2/3+			
	102410	Salted peanuts	555/1/2/300, ½ /1/2/3+			
	102450	Seeds	555/1/2/300, ½ /1/2/3+			
	102440	Unsalted nuts	555/1/2/300, ½ /1/2/3+			
	102420	Unsalted peanuts	555/1/2/300, ½ /1/2/3+			
	103310	Spreads/sauces consumers (dep)	0/1, N/Y			
	20088	Types of spreads/sauces consumed				
		Peanut butter	334 (assumed 1 serving)			
	Used mean intake across diet records for each item then summed item means for daily servings. Applied points			<0.5/wk	0.5-4/wk	>4/wk

Olive oil	20090	Type of fat/oil used in cooking yesterday (list of 29, check any, y/n)<br>Olive oil<br><br>*did not include olive oil-based spreads/margarines since these contain only 20-30% olive oil.	 <br>353			
	Olive oil is primary cooking oil (pt_molive=1) if participant used any olive oil in cooking across all diet records (regardless of whether they reported use of other fats in cooking as well; 20090 is not exclusive variable), otherwise pt_molive=0			No		Yes
Butter/margarine	103990<br>104030<br>104040<br><br>101300<br>20087	Vegetable consumers (dep)<br>Boiled/baked potatoes intake (dep)<br>Butter/margarine added to potatoes<br><br>But/marg on brd/crack (dep)<br>Types of spread used on brd/crack<br>Butter<br>Spreadable butter<br>Low fat butter<br>Normal fat butter<br>Unknown type of butter<br>Olive spread<br>Very low fat olive spread<br>Low fat olive spread<br>Normal fat olive spread<br>Cholesterol-lowering olive spread<br>Unknown olive spread<br>Polyunsaturated margarine<br>Very low fat polyunsaturated margarine<br>Low fat polyunsaturated margarine<br>Normal fat polyunsaturated margarine<br>Cholesterol-lowering polyunsaturated margarine<br>Unknown polyunsaturated margarine<br>Soya margarine<br>Very low fat soya margarine<br>Low fat soya margarine	0/1, N/Y<br>444/555/1/2/300, ¼ / ½ /1/2/3+<br>0/1, N/Y<br><br>0/1, N/Y<br><br>159<br>160<br>161<br>162<br>163<br>164<br>165<br>166<br>167<br>168<br>169<br>170<br>171<br>172<br>173<br>174<br>175<br>182<br>183<br>184<br>185			

		Normal fat soya margarine<br>Cholesterol-lowering soya margarine<br>Unknown soya margarine<br>Unknown margarine<br>Very low fat unknown margarine<br>Low fat unknown margarine<br>Unknown fat unknown margarine<br>Cholesterol lowering unknown margarine<br>Unknown margarine<br>Hard margarine<br><br>Did not consider data concerning thickness of spread on bread or whether butter/margarine was low-fat	186<br>187<br>188<br>189<br>190<br>191<br>192<br>193<br>194			
	Since 20087 is not an exclusive variable, we derived a “servings of butter/marg on bread” variable weighted by response (i.e if a non-butter/margarine spread and butter/margarine used, assumed 0.5*servings of bread with butter/marg). Assumed 1 serving (1 pat/tsp) butter/marg per serving of bread or potato. Used mean intake across diet records for each item (butter/marg on potatoes and bread) then summed item means for daily servings. Applied (reverse) points.			<1/d	1-2/d	>2/d
Cheese	102800<br>102820<br>102830<br>102840<br>102860<br>102870<br>102880<br>102890<br>102900<br>102910	Cheese consumers (dep)<br>Hard cheese<br>Soft cheese<br>Blue cheese<br>Cheese spread<br>Cottage cheese<br>Feta<br>Mozzarella<br>Goat's cheese<br>Other cheese<br>*did not use low fat versions	0/1, N/Y<br>555/1/2/300, ½ /1/2/3+<br>555/1/2/300, ½ /1/2/3+<br>555/1/2/300, ½ /1/2/3+<br>555/1/2/300, ½ /1/2/3+<br>555/1/2/300, ½ /1/2/3+<br>555/1/2/300, ½ /1/2/3+<br>555/1/2/300, ½ /1/2/3+<br>555/1/2/300, ½ /1/2/3+<br>555/1/2/300, ½ /1/2/3+			
	Used mean intake across diet records for each item then summed item means for daily servings. Applied (reverse) points			6+/wk	1<6/wk	<1/wk
Whole grains	100760<br>100770	Breakfast cereal consumer (dep)<br>Porridge	0/1, N/Y<br>555/1/200, ½ /1/2+			

	100800<br>100840<br>100850<br><br>100940<br>100950<br>20091<br>101020<br>20092<br>101090<br>20093<br>101160<br>20094<br>101250<br>101260<br><br>102700<br>102720<br>102740<br>102780	Muesli<br>Bran cereal<br>Whole-wheat cereal<br><br>Bread consumer (dep)<br>Sliced bread (dep)<br>Type of sliced bread eaten (>1 option)<br>Baguette (dep)<br>Type of baguette eaten (>1 option)<br>Bap (dep)<br>Type of large bap eaten (>1 option)<br>Bread roll (dep)<br>Type of bread roll eaten (>1 option)<br>Crispbread<br>Oatcakes<br><br>Starchy food consumer (dep)<br>Wholemeal pasta<br>Brown rice<br>Other grain (“bulgar, wheat, millett, barley”)	555/1/200, ½ /1/2+<br>555/1/200, ½ /1/2+<br>555/1/200, ½ /1/2+<br><br>0/1, N/Y<br>555/1/2/3/4/5/600, ½ /1/2/3/4/5/6+<br>1/2/3/4/5, whi/mix/ww/seed/oth<br>555/1/2/3/4/5/600, ½ /1/2/3/4/5/6+<br>1/2/3/4/5, whi/mix/ww/seed/oth<br>555/1/2/3/4/5/600, ½ /1/2/3/4/5/6+<br>1/2/3/4/5, whi/mix/ww/seed/oth<br>555/1/2/3/4/5/600, ½ /1/2/3/4/5/6+<br>1/2/3/4/5, whi/mix/ww/seed/oth<br>555/1/2/3/4/5/600, ½ /1/2/3/4/5/6+<br>555/1/2/3/4/5/600, ½ /1/2/3/4/5/6+<br><br>0/1, N/Y<br>555/1/200, ½ /1/2+<br>555/1/200, ½ /1/2+<br>555/1/200, ½ /1/2+			
	Since 20091, 20092, 20093, 20094 are not exclusive, we derived a “servings of whole-wheat bread” variable weighted by response. Used mean intake across diet records for each item then summed item means for daily servings. Applied points			<1/d	1-2/d	>2/d
Fish (not fried)	103140<br>103150<br>103160<br>103190<br>103200<br>103210<br>103220<br>103230	Fish consumer (dep)<br>Tinned tuna<br>Oily fish<br>White fish<br>Prawns<br>Lobster/crab<br>Shellfish<br>Other fish	0/1, N/Y<br>555/1/2/3/400, ½ /1/2/3/4+<br>555/1/2/3/400, ½ /1/2/3/4+<br>555/1/2/3/400, ½ /1/2/3/4+<br>555/1/2/3/400, ½ /1/2/3/4+<br>555/1/2/3/400, ½ /1/2/3/4+<br>555/1/2/3/400, ½ /1/2/3/4+<br>555/1/2/3/400, ½ /1/2/3/4+			
	Used mean intake across diet records for each item then summed item means for daily servings. Applied points			<1/m	1+/m	1+/wk
Beans	103990<br>104000	Vegetable consumers (dep)<br>Baked bean	0/1, N/Y<br>444/555/1/2/300, ¼ / ½ /1/2/3+			

	104010	Pulses	444/555/1/2/300, ¼ / ½ /1/2/3+			
	104110	Broad bean	444/555/1/2/300, ¼ / ½ /1/2/3+			
	102520	Soup consumer (dep)	0/1, N/Y			
	102540	Canned (dep)	555/1/2/3/400, ½ /1/2/3/4+			
	102620	Homemade (dep)	555/1/2/3/400, ½ /1/2/3/4+			
	20108	Ingredients in canned soup (>1 option)	4, pulses			
	20109	Ingredients in homemade soup (>1 option)<br>*1 bean soup= 0.25 bean serving	4, pulses			
	103310	Spreads/sauces consumers (dep)	0/1, N/Y			
	20088	Types of spreads/sauces consumed<br>Hummus	 336			
	103250	Vegetarian alternatives intake (dep)	0/1, N/Y			
	103270	Tofu intake	555/1/2/3/400, ½ /1/2/3/4+			
	Used mean intake across diet records for each item then summed item means for daily servings. Applied points			<1	1-3/wk	>3/wk
Poultry (not fried)	103000	Meat consumers (dep)	0/1, N/Y			
	103060	Poultry intake (dep)	555/1/2/3/4/500, ½ /1/2/3/4/5+			
	Used mean intake across diet records for “mpoultry”. Applied points			<1/wk	1/wk	2+/wk
Red meat & products	103000	Meat consumers (dep)	0/1, N/Y			
	103010	Sausage	555/1/2/3/4/500, ½ /1/2/3/4/5+			
	103020	Beef	555/1/2/3/4/500, ½ /1/2/3/4/5+			
	103030	Pork	555/1/2/3/4/500, ½ /1/2/3/4/5+			
	103040	Lamb	555/1/2/3/4/500, ½ /1/2/3/4/5+			
	103070	Bacon	555/1/2/3/4/500, ½ /1/2/3/4/5+			
	103080	Ham	555/1/2/3/4/500, ½ /1/2/3/4/5+			
	Used mean intake across diet records for each item then summed item means for daily servings. Applied (reverse) points			>6/wk	4-6/wk	<4/wk
Fast/fried foods	103000	Meat consumers (dep)	0/1, N/Y			
	103050	Crumbed or deep-fried poultry	555/1/2/3/4/500, ½ /1/2/3/4/5+			
	103140	Fish consumer (dep)	0/1, N/Y			
	103170	Breaded fish	555/1/2/3/400, ½ /1/2/3/4+			
	103180	Battered fish	555/1/2/3/400, ½ /1/2/3/4+			

	103990	Vegetable consumers (dep)	0/1, N/Y			
	104020	Fried potatoes	444/555/1/2/300, ¼ / ½ /1/2/3+			
	102400	Savoury snack consumers (dep)	0/1, N/Y			
	102460	Crisp/chips	555/1/2/300, ½ /1/2/3+			
		*did not include ‘pizza’ : "How many medium slices of pizza did you have? N.B. Fillings and toppings should be recorded later."<br>Pizza type unclear and may have been “double counting” with other MIND items above.				
	Used mean intake across diet records for each item then summed item means for daily servings. Applied (reverse) points			>3/wk	1-3/wk	<1/wk
Pastries and sweets	101970	Double crust pastry	555/1/2/3/400, ½ /1/2/3/4+			
	101980	Single crust pastry	555/1/2/3/400, ½ /1/2/3/4+			
	101990	Crumble	555/1/2/3/400, ½ /1/2/3/4+			
	102010	Pancake	555/1/2/3/400, ½ /1/2/3/4+			
	102020	Scotch pancake	555/1/2/3/400, ½ /1/2/3/4+			
	102030	Yorkshire pudding	555/1/2/3/400, ½ /1/2/3/4+			
	102050	Croissant	555/1/2/3/400, ½ /1/2/3/4+			
	102060	Danish pastry	555/1/2/3/400, ½ /1/2/3/4+			
	102070	Scone	555/1/2/3/400, ½ /1/2/3/4+			
	102080	Yogurt/ice-cream consumers (dep)	0/1, N/Y			
	102120	Ice-cream	555/1/2/300, ½ /1/2/3+			
	102130	Dessert consumers (dep)	0/1, N/Y			
	102140	Milk-based pudding	555/1/2/300, ½ /1/2/3+			
	102150	Other milk-based pudding	555/1/2/300, ½ /1/2/3+			
	102170	Soya dessert	555/1/2/300, ½ /1/2/3+			
	102180	Fruitcake	555/1/2/300, ½ /1/2/3+			
	102190	Cake	555/1/2/300, ½ /1/2/3+			
	102200	Doughnut	555/1/2/300, ½ /1/2/3+			
	102210	Sponge pudding	555/1/2/300, ½ /1/2/3+			

	102220<br>102230<br><br>102250<br>102260<br>102270<br>102280<br>102290<br>102300<br>102310<br>102330<br>102340<br>102350<br>102360<br>102370<br>102380<br><br>100530<br>100550<br>100170<br>100180<br><br>26064	Cheesecake<br>Other dessert<br><br>Sweet snack consumers (dep)<br>Chocolate bar<br>White chocolate<br>Milk chocolate<br>Dark chocolate<br>Chocolate-covered raisin<br>Chocolate sweet<br>Sweets<br>Chocolate-covered biscuits<br>Chocolate biscuits<br>Sweet biscuits<br>Cereal bar<br>Other sweets<br><br>Flavored milk intake<br>Hot chocolate intake<br>Fizzy drink intake<br>Squash intake<br><br>Added sugars and preserves: derived from UKB diet committee and includes table sugar added to coffee, tea and cereal (semi-quantified), as well as jam, honey and syrup (binary).	555/1/2/300, ½ /1/2/3+<br>555/1/2/300, ½ /1/2/3+<br><br>0/1, N/Y<br>444/555/1/2/3/4/500, ¼ / ½ /1/2/3/4/5+<br>444/555/1/2/3/4/500, ¼ / ½ /1/2/3/4/5+<br>444/555/1/2/3/4/500, ¼ / ½ /1/2/3/4/5+<br>444/555/1/2/3/4/500, ¼ / ½ /1/2/3/4/5+<br>444/555/1/2/3/4/500, ¼ / ½ /1/2/3/4/5+<br>444/555/1/2/3/4/500, ¼ / ½ /1/2/3/4/5+<br>444/555/1/2/3/4/500, ¼ / ½ /1/2/3/4/5+<br>444/555/1/2/3/4/500, ¼ / ½ /1/2/3/4/5+<br>444/555/1/2/3/4/500, ¼ / ½ /1/2/3/4/5+<br>444/555/1/2/3/4/500, ¼ / ½ /1/2/3/4/5+<br>444/555/1/2/3/4/500, ¼ / ½ /1/2/3/4/5+<br>444/555/1/2/3/4/500, ¼ / ½ /1/2/3/4/5+<br><br>555/1/2/3/4/5/600, ½ /1/2/3/4/5/6+<br>555/1/2/3/4/5/600, ½ /1/2/3/4/5/6+<br>555/1/2/3/4/5/600, ½ /1/2/3/4/5/6+<br>555/1/2/3/4/5/600, ½ /1/2/3/4/5/6+<br><br>g<br>to align this variable with other sweet foods, assumed 1 serving= 15 g			
	Used mean intake across diet records for each item then summed item means for daily servings. Applied (reverse) points			7+/wk	5-6/wk	<5/wk
Wine	100580<br>100590<br>20096<br>100630<br>20097<br>100670<br>20095<br>100720	Alcohol consumed (dep)<br>Red wine<br>Size of red wine glass drunk (>1 option)<br>Rose wine<br>Size of rose wine glass drunk (>1 option)<br>White wine<br>Size of white wine glass drunk (>1 option)<br>Fortified wine (includes sherry, port)	0/1, N/Y<br>555/1/2/3/4/5/600, ½ /1/2/3/4/5/6+<br>1/2/3, 125/175/250ml<br>555/1/2/3/4/5/600, ½ /1/2/3/4/5/6+<br>1/2/3, 125/175/250ml<br>555/1/2/3/4/5/600, ½ /1/2/3/4/5/6+<br>1/2/3, 125/175/250ml<br>555/1/2/3/4/5/600, ½ /1/2/3/4/5/6+			

	5 oz servings per day. If multiple glass sizes, derived mean. Missing glass size assumed 175ml (mode). Used mean intake across diet records for each item then summed item means for daily servings. Applied (reverse) points	<1/m	1/m-1/wk	2-7/wk

Dep: dependent variable (determines whether another question was asked); N: no; Y: yes.

Supplementary Table S2. AHEI score derivation in UK Biobank.

Diet component	UKB variable#	Label	Coding (standard servings per day)	Min*	Max
				0	10
Vegetables	103990	Veg consumer (dep)	0/1, N/Y		
	104160	Cabbage/kale	444/555/1/2/300, ¼ / ½ /1/2/3+		
	104240	Lettuce	444/555/1/2/300, ¼ / ½ /1/2/3+		
	104300	Spinach	444/555/1/2/300, ¼ / ½ /1/2/3+		
	104370	Watercress	444/555/1/2/300, ¼ / ½ /1/2/3+		
	104080	Coleslaw (cabbage)	444/555/1/2/300, ¼ / ½ /1/2/3+		
	104090	Side salad	444/555/1/2/300, ¼ / ½ /1/2/3+		
	104060	Mixed veg	444/555/1/2/300, ¼ / ½ /1/2/3+		
	104070	Veg pieces	444/555/1/2/300, ¼ / ½ /1/2/3+		
	104120	Green beans	444/555/1/2/300, ¼ / ½ /1/2/3+		
	104130	Beet root	444/555/1/2/300, ¼ / ½ /1/2/3+		
	104140	Broccoli	444/555/1/2/300, ¼ / ½ /1/2/3+		
	104150	Butternut squash	444/555/1/2/300, ¼ / ½ /1/2/3+		
	104170	Carrot	444/555/1/2/300, ¼ / ½ /1/2/3+		
	104180	Cauliflower	444/555/1/2/300, ¼ / ½ /1/2/3+		
	104190	Celery	444/555/1/2/300, ¼ / ½ /1/2/3+		
	104200	Courgetti/zuch	444/555/1/2/300, ¼ / ½ /1/2/3+		
	104210	Cucumber	444/555/1/2/300, ¼ / ½ /1/2/3+		
	104220	Garlic	444/555/1/2/300, ¼ / ½ /1/2/3+		
	104230	Leek	444/555/1/2/300, ¼ / ½ /1/2/3+		
	104250	Mushroom	444/555/1/2/300, ¼ / ½ /1/2/3+		
	104260	Onion	444/555/1/2/300, ¼ / ½ /1/2/3+		
	104270	Parsnip	444/555/1/2/300, ¼ / ½ /1/2/3+		
	104280	Pea (frozen, fresh, tinned)	444/555/1/2/300, ¼ / ½ /1/2/3+		
	104290	Sweet pepper	444/555/1/2/300, ¼ / ½ /1/2/3+		
	104310	Sprouts	444/555/1/2/300, ¼ / ½ /1/2/3+		
	104320	Sweetcorn	444/555/1/2/300, ¼ / ½ /1/2/3+		
	104330	Sweet potato	444/555/1/2/300, ¼ / ½ /1/2/3+		
	104340	Fresh tomato	444/555/1/2/300, ¼ / ½ /1/2/3+		
	104350	Tinned tomato	444/555/1/2/300, ¼ / ½ /1/2/3+		
	104360	Turnip/swede	444/555/1/2/300, ¼ / ½ /1/2/3+		
	104380	Other veg	444/555/1/2/300, ¼ / ½ /1/2/3+		
	102520	Soup consumer (dep)	0/1, N/Y		

	102540<br>102620<br>20108<br>20109<br><br>103310<br>20088	Canned (dep)<br>Homemade (dep)<br>Ingredients in canned soup<br>Ingredients in homemade soup<br><br>Spreads/sauces consumers (dep)<br>Types of spreads/sauces consumed<br>Tomato sauce<br><br>*1veg soup= 0.25 veg serving	555/1/2/3/400, ½ /1/2/3/4+<br>555/1/2/3/400, ½ /1/2/3/4+<br>5, vegetables<br>5, vegetables<br><br>0/1, N/Y<br><br>346 (max 1 serving)		
	Used mean intake across diet records for each item then summed item means for daily servings. Applied points			0/d	≥5/d
Fruit (not juice)	104400<br>104410<br>104420<br>104430<br>104440<br>104450<br>104460<br>104470<br>104480<br>104490<br>104500<br>104510<br>104520<br>104530<br>104540<br>104550<br>104560<br>104570<br>104580<br>104590<br>104100	Fruit consumer (dep)<br>Stewed fruit intake<br>Prune intake<br>Dried fruit intake<br>Mixed fruit intake<br>Apple intake<br>Banana intake<br>Berry intake<br>Cherry intake<br>Grapefruit intake<br>Grape intake<br>Mango intake<br>Melon intake<br>Orange intake<br>Satsuma intake<br>Peach/nectarine intake<br>Pear intake<br>Pineapple intake<br>Plum intake<br>Other fruit intake<br>Avocado	0/1, N/Y<br>555/1/2/3/400, ½ /1/2/3/4+		
	Used mean intake across diet records for each item then summed item means for daily servings. Applied points			0/d	≥4/d
Whole grains	26076<br>26077	Bran cereal<br>Oat cereal (non sugar)	g/d<br>g/d		

(modified per Wang et al 2014)	26074<br>26114<br><br>26105	Wholemeal bread<br>Wholemeal pasta, brown rice and other wholegrains<br>Muesli (1/2 serving)	g/d<br>g/d<br>g/d<br>g/d		
	Used derived food weight variables available to UKB researchers to determine total g of wholegrain foods; not whole grain g per se. Assumed whole grains contributed half the weight of Muesli (fruit/nuts the other half).			0/d	75 g/d (F)<br>90 g/d (M)
Sugar sweetened beverages + fruit juice	100530<br>100550<br>100170<br>100180<br>100190<br>100200<br>100220<br>100210	Flavored milk intake<br>Hot chocolate intake<br>Fizzy drink intake<br>Squash intake<br>Orange juice intake<br>Grapefruit juice intake<br>Fruit smoothie intake<br>Pure fruit/vegetable juice intake (1 serving=1/2 fruit juice serving)	555/1/2/3/4/5/600, ½ /1/2/3/4/5/6+<br>555/1/2/3/4/5/600, ½ /1/2/3/4/5/6+<br>555/1/2/3/4/5/600, ½ /1/2/3/4/5/6+<br>555/1/2/3/4/5/600, ½ /1/2/3/4/5/6+<br>555/1/2/3/4/5/600, ½ /1/2/3/4/5/6+<br>555/1/2/3/4/5/600, ½ /1/2/3/4/5/6+<br>555/1/2/3/4/5/600, ½ /1/2/3/4/5/6+<br>555/1/2/3/4/5/600, ½ /1/2/3/4/5/6+		
	Used mean intake across diet records for each item then summed item means for daily intake. Applied (reverse) points.			≥1/d	0
Nuts & Legumes	103990<br>104000<br>104010<br>104110<br><br>102520<br>102540<br>102620<br>20108<br>20109<br><br><br>103310<br>20088<br><br><br><br>103250<br>103270	Vegetable consumers (dep)<br>Baked bean<br>Pulses<br>Broad bean<br><br>Soup consumer (dep)<br>Canned (dep)<br>Homemade (dep)<br>Ingredients in canned soup (>1 option)<br>Ingredients in homemade soup (>1 option)<br>*1 bean soup= 0.25 bean serving<br><br>Spreads/sauces consumers (dep)<br>Types of spreads/sauces consumed<br>Hummus<br>Peanut butter<br><br>Vegetarian alternatives intake (dep)<br>Tofu intake	0/1, N/Y<br>444/555/1/2/300, ¼ / ½ /1/2/3+<br>444/555/1/2/300, ¼ / ½ /1/2/3+<br>444/555/1/2/300, ¼ / ½ /1/2/3+<br><br>0/1, N/Y<br>555/1/2/3/400, ½ /1/2/3/4+<br>555/1/2/3/400, ½ /1/2/3/4+<br>4, pulses<br>4, pulses<br><br><br>0/1, N/Y<br><br>336<br>334 (assumed 1 serving)<br><br>0/1, N/Y<br>555/1/2/3/400, ½ /1/2/3/4+		

	102400<br>102430<br>102410<br>102450<br>102440<br>102420	Savory snack consumer (dep)<br>Salted nuts<br>Salted peanuts<br>Seeds<br>Unsalted nuts<br>Unsalted peanuts	0/1, N/Y<br>555/1/2/300, ½ /1/2/3+<br>555/1/2/300, ½ /1/2/3+<br>555/1/2/300, ½ /1/2/3+<br>555/1/2/300, ½ /1/2/3+<br>555/1/2/300, ½ /1/2/3+		
	Used mean intake across diet records for each item then summed item means for daily servings. Applied points			0/d	≥1/d
Red meat & products	103000<br>103010<br>103020<br>103030<br>103040<br>103070<br>103080	Meat consumers (dep)<br>Sausage<br>Beef<br>Pork<br>Lamb<br>Bacon<br>Ham	0/1, N/Y<br>555/1/2/3/4/500, ½ /1/2/3/4/5+<br>555/1/2/3/4/500, ½ /1/2/3/4/5+<br>555/1/2/3/4/500, ½ /1/2/3/4/5+<br>555/1/2/3/4/500, ½ /1/2/3/4/5+<br>555/1/2/3/4/500, ½ /1/2/3/4/5+<br>555/1/2/3/4/500, ½ /1/2/3/4/5+		
	Used mean intake across diet records for each item then summed item means for daily servings. Applied (reverse) points			≥1.5/d	0
Trans fat, %energy	26155<br>26002	Trans fatty acids<br>Energy, kj	g/d<br>kj/d		
	Converted trans g to calories and derived % of total energy (cal=kj*0.2388458966275). Used mean intake across diet records. Applied (reverse) points.			≥4	≤0.5
Long chain (n-3) fats (EPA +DHA), mg/d	103140<br>103160	Fish consumer (dep)<br>Oily fish	0/1, N/Y<br>555/1/2/3/400, ½ /1/2/3/4+		
	The cutoff for optimal intake per ref (250 mg/d) is ~2 4-oz servings of fish /wk. Per Perez et al, a serving of fish was assumed 100g (3.6 oz). We applied the cutoff for optimal intake as 2 servings per week.			0	≥2/wk
PUFA, % energy	26015<br>26016<br>26002	n-3 fatty acids<br>n-6 fatty acids<br>Energy, kj	g/d<br>g/d<br>kj/d		
	Summed n-3 and n-6 and converted g to calories and derived % of total energy (cal=kj*0.2388458966275). Used mean intake across diet records. This component includes EPA + DHA but contributes minimally to score. Applied points.			≤2	≥10
Sodium, mg/d	26052	Sodium	mg/d		
	Used mean derived nutrient intake across diet records.			Highest decile	Lowest decile
Alcohol, drinks/d	26067	Beer and cider	g/d	≥2.5 (F)	0.5-1.5 (F)

	26151<br>26152<br>26153<br>26138	Fortified wine<br>Red wine<br>White wine<br>Spirits	g/d<br>g/d<br>g/d<br>g/d	≥3.5 (F)	0.5-2.0 (M)
	Used derived food weight variables available to UKB researchers. One drink is 4 oz of wine, 12 oz of beer, or 1.5 oz of spirits (1 oz = 28.35 g)				

*A score of 10 indicates that the recommendations were fully met, whereas a score of 0 represents the least healthy dietary behavior. Intermediate intakes were scored proportionately between 0 and 10. EPA: eicosapentaenoic acid; DHA: docosahexaenoic acid; PUFA: polyunsaturated fatty acids; F: female; M: male; dep: dependent variable (determines whether another question was asked); N: no; Y: yes.

Supplementary Table S3. MIND components in UK Biobank.

Component	Score	*n*	%	*n*	%	*n*	%
Green leafy vegetables	0	27,940	69.4	17,618	49.0	13,067	29.4
	0.5	10,313	25.6	13,843	38.5	18,680	42.0
	1	2003	5.0	4502	12.5	12,695	28.6
Other vegetables	0	11,113	27.6	3259	9.1	1198	2.7
	0.5	3454	8.6	2153	6.0	1293	2.9
	1	25,689	63.8	30,551	85.0	41,951	94.4
Berries	0	34,463	85.6	24,976	69.5	20,226	45.5
	0.5	2350	5.8	34,93	9.7	5248	11.8
	1	3443	8.6	74,94	20.8	18,968	42.7
Nuts	0	32,369	80.4	24,217	67.3	22,475	50.6
	0.5	6093	15.1	8575	23.8	13,637	30.7
	1	1794	4.5	3171	8.8	8330	18.7
Olive oil	0	38,358	95.3	31,674	88.1	32,168	72.4
	1	1898	4.7	4289	11.9	12,274	27.6
Butter/margarine	0	13,578	33.7	8207	22.8	5985	13.5
	0.5	16,766	41.7	14,545	40.4	15,397	34.7
	1	9912	24.6	13,211	36.7	23,060	51.9
Cheese (not low fat)	0	6827	17.0	4507	12.5	4126	9.3
	0.5	21,118	52.5	19,056	53.0	22,572	50.8
	1	12,311	30.6	12,400	34.5	17,744	39.9
Whole grains	0	19,104	47.5	11,681	32.5	9543	21.5
	0.5	14,067	34.9	14,796	41.1	18,858	42.4
	1	7085	17.6	9486	26.4	16,041	36.1
Fish (not fried)	0	28,409	70.6	16,582	46.1	11,620	26.2
	0.5	613	1.5	714	2.0	649	1.5
	1	11,234	27.9	18,667	51.9	32,173	72.4
Beans	0	30,111	74.8	22,486	62.5	20,888	47.0
	0.5	6211	15.4	7474	20.8	10,449	23.5
	1	3934	9.8	6003	16.7	13,105	29.5
Poultry (not fried)	0	26,728	66.4	18,144	50.5	17,308	39.0
	0.5	4456	11.1	4640	12.9	5616	12.6
	1	9072	22.5	13,179	36.7	21,518	48.4
Red meat & products	0	25,479	63.3	14,853	41.3	9977	22.5
	0.5	4161	10.3	5042	14.0	5517	12.4
	1	10,616	26.4	16,068	44.7	28,948	65.1
Fast/fried foods	0	25,089	62.3	14,590	40.6	9532	21.5
	0.5	6857	17.0	8448	23.5	10,081	22.7
	1	8310	20.6	12,925	35.9	24,829	55.9
Pastries and sweets	0	37,942	94.3	31,799	88.4	33,458	75.3
	0.5	820	2.0	1312	3.7	2785	6.3
	1	1494	3.7	2852	7.9	8199	18.5
Wine	0	34,949	86.8	26,778	74.5	26,930	60.6
	0.5	250	0.6	358	1.0	549	1.2
	1	5057	12.6	8827	24.5	16,963	38.2

Supplementary Table S4. Baseline characteristics of participants 55+ years of age by tertiles (T) of MIND and AHEI-2010 score[1]

Characteristic	MIND			AHEI−2010		
	T1<br>0.0–5.5<br>*n* = 24,887	T2<br>5.5–7.0<br>*n* = 30,700	T3<br>7.5–14.5<br>*n* = 23,076	T1<br>12.2–51.0<br>*n* = 26,221	T2<br>51.0–63.2<br>*n* = 26,221	T3<br>63.2–107.1<br>*n* = 26,221
Age, years	62.7±4.4	62.8±4.3	62.6±4.3	62.6±4.4	62.8±4.3	62.8±4.3
Female, *n* (%)	9977 (40.1)	16,909 (55.1)	15,672 (67.9)	10,855 (41.4)	14,465 (55.2)	17,238 (65.7)
Race/ethnicity, *n* (%)						
White	24,495 (98.4)	30,055 (97.9)	22,404 (97.1)	25,807 (98.4)	25,685 (98.0)	25,462 (97.1)
South Asian	95 (0.4)	191 (0.6)	191 (0.8)	90 (0.3)	153 (0.6)	234 (0.9)
Black	47 (0.2)	100 (0.3)	113 (0.5)	61 (0.2)	68 (0.3)	131 (0.5)
Chinese	27 (0.1)	52 (0.2)	64 (0.3)	29 (0.1)	40 (0.2)	74 (0.3)
Other, Unknown	223 (0.90)	302 (1.0)	304 (1.3)	234 (0.9)	275 (1.0)	320 (1.2)
Townsend	−1.84±2.75	−1.92±2.70	−1.80±2.74	−1.84±2.74	−1.90±2.70	−1.84±2.73
Household income 52000+, *n* (%)	5088 (20.4)	7126 (23.2)	6027 (26.1)	6188 (23.6)	5906 (22.5)	6147 (23.4)
College/university degree, *n* (%)	9253 (37.2)	13,563 (44.2)	11,931 (51.7)	10410 (39.7)	11456 (43.7)	12881 (49.1)
Currently employed, *n* (%)	11,494 (46.2)	13,862 (45.2)	10,720 (46.5)	12,255 (46.7)	11,789 (45.0)	12032 (45.9)
Current smoker, *n* (%)	2130 (8.6)	1712 (5.6)	898 (3.9)	2311 (8.8)	1495 (5.7)	934 (3.6)
BMI, kg/m^2	27.6±4.6	26.8±4.4	26.1±4.3	27.7±4.6	26.9±4.4	25.9±4.2
Moderate to vigorous physical activity, hours/week	70.4±88.4	73.1±82.7	77.1±81.8	68.9±85.3	73.4±83.9	78.0±83.7
Self−reported health, *n* (%)						
Excellent	4280 (17.2)	6505 (21.2)	5574 (24.2)	4664 (17.9)	5441 (20.8)	6254 (23.9)
Good	14,908 (60.0)	18,784 (61.3)	14,131 (61.3)	15743 (60.1)	16009 (61.2)	16071 (61.4)
Fair	4806 (19.4)	4669 (15.2)	2925 (12.7)	4879 (18.6)	4120 (15.7)	3401 (13.0)
Poor	849 (3.4)	683 (2.2)	406 (1.8)	891 (3.4)	604 (2.3)	443 (1.7)
Family history of dementia, *n* (%)	592 (2.4)	817 (2.7)	636 (2.8)	644 (2.5)	705 (2.7)	696 (2.7)
Diabetes, *n* (%)	1004 (4.0)	931 (3.0)	519 (2.3)	949 (3.6)	834 (3.2)	671 (2.6)
Heart disease, *n* (%)	1602 (6.4)	1575 (5.1)	939 (4.1)	1625 (6.2)	1362 (5.2)	1129 (4.3)
Hypertension, *n* (%)	5592 (22.5)	6244 (20.3)	3986 (17.3)	5956 (22.7)	5275 (20.1)	4591 (17.5)
Stroke, *n* (%)	396 (1.6)	401 (1.3)	238 (1.0)	429 (1.6)	333 (1.3)	273 (1.0)
Depression, *n* (%)	1056 (4.2)	1123 (3.7)	870 (3.8)	1048 (4.0)	985 (3.8)	1016 (3.9)
Apoe ε4 carriers, *n* (%)[2]	5676 (27.2)	7248 (27.9)	5412 (27.6)	5886 (26.7)	6129 (27.7)	6321 (28.5)
GS_{AD}[2]	26.9±3.0	26.9±3.1	26.9±3.1	26.9±3.1	26.9±3.0	26.8±3.1
Energy, calories/d	2077±434	2001±423	1944±421	2090±436	1988±425	1947±413
Frequent fast meal consumer, %	759 (3.1)	619 (2.0)	345 (1.5)	824 (3.1)	541 (2.1)	358 (1.4)

Vitamin/mineral supplement user, %	11,239 (45.2)	15,865 (51.7)	13,371 (57.9)	11937 (45.5)	13466 (51.4)	15072 (57.5)

[1]Values are mean±SD or *n* (%). Age at the time of first Oxford WebQ. Non-diet data collected at baseline assessment center visit or linked hospital records. [2]*n* = 20899, 25941, 19582 for MIND T1, T2 and T3, respectively. *n* = 22057, 22153, 22212 for AHEI T1, T2 and T3, respectively

Supplementary Table S5. AHEI-2010 components in UK Biobank.

Components	Mean Score	SD
Vegetables	5.1	2.9
Fruit (not juice)	5.2	3.1
Whole grains	5.9	4.1
Sugar sweetened beverages + fruit juice	4.8	4.0
Nuts & Legumes	3.4	3.5
Red meat & products	5.2	3.6
Trans fat	9.8	0.4
Long chain (*n*–3) fats (EPA +DHA)	2.6	4.2
PUFA	4.5	1.9
Sodium	5.0	3.2
Alcohol	4.9	3.6
Total	62.7	12.5

Supplementary Table S6 Associations between adherence to MIND and cognitive ability with and without adjustment for education and income level.

	n	Test Score[3]	Model 1 plus education & income[1]		Model 2 without education & income[2]	
			β (95% CI)	*P*	β (95% CI)	*P*
			[4]Fluid Intelligence			
MIND T1	18764	6.69 (2.07)	Reference		Reference	
MIND T2	16865	6.73 (2.05)	−0.04 (−0.08, −0.001)	0.04	0.09 (0.05, 0.13)	<0.0001
MIND T3	21003	6.69 (2.02)	−0.16 (−0.20, −0.12)	<0.0001	0.10 (0.06, 0.14)	<0.0001
MIND score (raw), linear trend			−0.04 (−0.05, −0.03)	<0.0001	0.02 (0.01, 0.03)	<0.0001
			[5]Reaction Time			
MIND T1	40084	542 (101)	Reference		Reference	
MIND T2	35807	544 (99)	−0.94 (−2.32, 0.43)	0.18	−1.44 (−2.82, −0.06)	0.04
MIND T3	44224	548 (101)	0.18 (−1.17, 1.52)	0.80	−1.31 (−2.66, 0.03)	0.06
MIND score (raw), linear trend			0.21 (−0.09, 0.50)	0.17	−0.16 (−0.45, 0.13)	0.29
			[5]Pairs Matching			
MIND T1	39941	1.38 (0.62)	Reference		Reference	
MIND T2	35609	1.39 (0.62)	0.01 (0.004, 0.02)	0.004	0.005 (−0.003, 0.01)	0.23
MIND T3	43999	1.42 (0.62)	0.04 (0.03, 0.04)	<0.0001	0.02 (0.01, 0.03)	<0.0001
MIND score (raw), linear trend			0.01 (0.008, 0.01)	<0.0001	0.006 (0.004, 0.008)	<0.0001
			[4]Symbol Digit Substitution			
MIND T1	23075	20.2 (5.1)	Reference		Reference	
MIND T2	21153	20.1 (5.1)	−0.04 (−0.12, 0.05)	0.37	0.04 (−0.04, 0.13)	0.35
MIND T3	26404	19.9 (5.0)	−0.23 (−0.32, −0.15)	<0.0001	−0.02 (−0.10, 0.06)	0.63
MIND score (raw), linear trend			−0.07 (−0.09, −0.05)	<0.0001	−0.02 (−0.03, 0.002)	0.08
			[5]Trail A			
MIND T1	20603	3.58 (0.32)	Reference		Reference	
MIND T2	18780	3.59 (0.32)	0.004 (−0.002, 0.01)	0.15	−0.0005 (−0.006, 0.005)	0.87
MIND T3	23258	3.60 (0.31)	0.01 (0.007, 0.02)	<0.0001	0.001 (−0.005, 0.007)	0.71
MIND score (raw), linear trend			0.003 (0.002, 0.004)	<0.0001	−0.00003 (−0.001, 0.001)	0.96
			[5]Trail B			
MIND T1	20603	4.10 (0.33)	Reference		Reference	
MIND T2	18780	4.12 (0.33)	0.009 (0.004, 0.02)	0.002	0.002 (−0.004, 0.008)	0.54
MIND T3	23257	4.13 (0.33)	0.02 (0.02, 0.03)	<0.0001	0.004 (−0.002, 0.01)	0.16
MIND score (raw), linear trend			0.006 (0.005, 0.007)	<0.0001	0.001 (0.0002, 0.003)	0.03
			[4]Prospective Memory Test			
	n	% correct	OR (95% CI)	*P*	OR (95% CI)	*P*
MIND T1	18887	86	Reference		Reference	
MIND T2	16978	86	1.00 (0.94, 1.07)	0.94	1.05 (0.99, 1.12)	0.12
MIND T3	21132	85	0.94 (0.88, 0.99)	0.03	1.04 (0.98, 1.10)	0.26
MIND score (raw), linear trend			0.98 (0.96, 0.99)	0.004	1.00 (0.99, 1.01)	0.85

[1]Model 1: adjusted for age, sex and self−reported race/ethnicity; [2]Model 2: adjusted for age, sex, race/ethnicity, [education, income], Townsend deprivation index, employment status, global cognition score, family history of dementia; history of hypertension, diabetes, heart disease, stroke and depression; self−reported health, smoking, physical activity, BMI, fast meal consumption and energy intake. [3]Data are raw mean (SD) scores of each cognitive function test. [4]Positive beta−coefficients for FI (difference in 13−point score) and SDS (difference in number of correct substitutions) and OR >1 for PM (correct on first attempt) correspond to higher performance compared to MIND tertile 1. [5]Negative beta−coefficients for Pairs (difference in (log−transformed) number of errors), RT (difference in time, seconds, to respond), Trails A and Trails B (difference in (log transformed) time to complete) correspond to higher performance compared to MIND tertile 1.

Supplementary Table S7 Associations between adherence to MIND and cognitive ability among participants aged 55+ years.

	n	Test Score[3]	Model 1[1]		Model 2[2]	
			β (95% CI)	*P*	β (95% CI)	*P*
[4]Fluid Intelligence						
MIND T1	11141	6.68 (2.06)	Reference		Reference	
MIND T2	13915	6.70 (2.01)	0.10 (0.05, 0.15)	0.0001	−0.05 (−0.10, −0.007)	0.02
MIND T3	10697	6.66 (1.98)	0.10 (0.05, 0.16)	0.0001	−0.17 (−0.22, −0.12)	<0.0001
MIND score (raw), linear trend			0.02 (0.01, 0.03)	0.0001	−0.04 (−0.05, −0.03)	<0.0001
MIND score (per SD), linear trend			0.04 (0.02, 0.06)		−0.08 (−0.10, −0.06)	
[5]Reaction Time						
MIND T1	24762	567 (103)	Reference		Reference	
MIND T2	30552	562 (101)	−2.75 (−4.46, −1.05)	0.002	−0.87 (−2.58, 0.84)	0.32
MIND T3	22957	566 (104)	−2.12 (−3.97, −0.27)	0.03	1.14 (−0.75, 3.04)	0.24
MIND score (raw), linear trend			−0.48 (−0.86, −0.10)	0.01	0.27 (−0.12, 0.66)	0.17
MIND score (per SD), linear trend			−0.92 (−1.65, −0.19)		0.52 (−0.22, 1.27)	
[5]Pairs Matching						
MIND T1	24626	1.44 (0.62)	Reference		Reference	
MIND T2	30340	1.45 (0.62)	0.01 (0.0007, 0.02)	0.04	0.01 (0.004, 0.03)	0.007
MIND T3	22787	1.46 (0.62)	0.02 (0.01, 0.04)	<0.0001	0.03 (0.02, 0.04)	<0.0001
MIND score (raw), linear trend			0.006 (0.004, 0.008)	<0.0001	0.007 (0.005, 0.01)	<0.0001
MIND score (per SD), linear trend			0.01 (0.007, 0.02)		0.01 (0.009, 0.02)	
[4]Symbol Digit Substitution						
MIND T1	14474	18.8 (4.8)	Reference		Reference	
MIND T2	18381	18.8 (4.8)	0.08 (−0.02, 0.18)	0.13	−0.11 (−0.21, −0.01)	0.03
MIND T3	13769	18.7 (4.8)	0.03 (−0.07, 0.14)	0.54	−0.28 (−0.39, −0.17)	<0.0001
MIND score (raw), linear trend			0.005 (−0.02, 0.03)	0.67	−0.07 (−0.09, −0.05)	<0.0001
MIND score (per SD), linear trend			0.009 (−0.03, 0.05)		−0.13 (−0.17, −0.09)	
[5]Trail A						
MIND T1	12757	3.64 (0.31)	Reference		Reference	
MIND T2	16078	3.65 (0.31)	−0.0006 (−0.008, 0.006)	0.86	0.007 (0.0002, 0.01)	0.04
MIND T3	11954	3.65 (0.30)	−0.005 (−0.01, 0.003)	0.21	0.009 (0.001, 0.02)	0.03
MIND score (raw), linear trend			−0.001 (−0.003, 0.0005)	0.16	0.002 (0.0005, 0.004)	0.01
MIND score (per SD), linear trend			−0.002 (−0.005, 0.0009)		0.004 (0.0009, 0.007)	
[5]Trail B						
MIND T1	12757	4.19 (0.33)	Reference		Reference	
MIND T2	16078	4.19 (0.32)	−0.005 (−0.01, 0.002)	0.17	0.01 (0.003, 0.02)	0.004
MIND T3	11953	4.20 (0.32)	−0.003 (−0.01, 0.005)	0.41	0.02 (0.02, 0.03)	<0.0001
MIND score (raw), linear trend			−0.0007 (−0.002, 0.001)	0.42	0.006 (0.004, 0.007)	<0.0001
MIND score (per SD), linear trend			−0.001 (−0.004, 0.002)		0.01 (0.008, 0.01)	
[4]Prospective Memory Test						
	n	% correct	OR (95% CI)	*P*	OR (95% CI)	*P*
MIND T1	11234	83.7	Reference		Reference	
MIND T2	11827	84.3	1.10 (1.03, 1.18)	0.008	1.05 (0.98, 1.12)	0.20
MIND T3	10771	83.4	1.06 (0.98, 1.14)	0.15	0.96 (0.89, 1.04)	0.35
MIND score (raw), linear trend			1.01 (0.99, 1.03)	0.24	0.99 (0.97, 1.00)	0.15
MIND score (per SD), linear trend			1.02 (0.99, 1.05)		0.98 (0.95, 1.01)	

[1]Model 1: adjusted for age, sex and self−reported race. [2]Model 2: adjusted for age, sex, race, smoking, Townsend deprivation index, education, income, employment status, self−reported health, BMI, physical activity, family history of dementia, non−home cooked food consumption, energy OTHERs. [3]Data are raw mean (SD) scores of each cognitive function test. [4]Positive beta−coefficients for FI (difference in 13−point score) and SDS (difference in number of correct substitutions) and OR >1 for PM (correct on first attempt) correspond to higher performance compared to MIND tertile 1. [5]Negative beta−coefficients for Pairs (difference in (log−transformed) number of errors), RT (difference in time,

seconds, to respond), Trails A and Trails B (difference in (log transformed) time to complete) correspond to higher performance compared to MIND tertile 1.

Supplementary Table S8. MIND score×Education (higher vs lower) interaction tests.

Cognition Test[1]	Definition of higher education			
	College/university		Level 4+ qualifications	
	Interaction β	*P*	Interaction β	*P*
FI	−0.05	<0.0001	−0.009	0.33
RT	0.53	0.06	0.85	0.009
Pairs	0.003	0.09	0.002	0.28
SDS	−0.07	0.0001	−0.04	0.04
Trail A	0.003	0.006	0.005	0.0006
Trail B	0.005	<0.0001	0.004	0.004
PM	−0.05	<0.0001	−0.07	<0.0001

[1]Higher FI, SDS and PM scores correspond to higher performance; lower RT, Pairs, Trail A and Trail B scores correspond to higher performance.

Supplementary Table S9. Associations between adherence to AHEI and cognitive ability among participants aged 55+ years.

	n	Test Score[3]	Model 1[1]		Model 2[2]	
			β (95% CI)	*P*	β (95% CI)	*P*
			[4]Fluid Intelligence			
AHEI T1	11780	6.77 (2.03)	Reference		Reference	
AHEI T2	11868	6.68 (2.02)	−0.03 (−0.08, 0.02)	0.24	−0.09 (−0.14, −0.04)	0.0003
AHEI T3	12105	6.59 (1.99)	−0.06 (−0.11, −0.010)	0.02	−0.21 (−0.26, −0.16)	<0.0001
AHEI score (raw), linear trend			−0.002 (−0.003, −0.0003)	0.02	−0.007 (−0.008, −0.006)	<0.0001
AHEI score (per SD), linear trend			−0.02 (−0.05, −0.004)		−0.10 (−0.12, −0.08)	
			[5]Reaction Time			
AHEI T1	26103	559 (102)	Reference		Reference	
AHEI T2	26095	563 (102)	0.31 (−1.43, 2.05)	0.73	1.30 (−0.45, 3.05)	0.15
AHEI T3	26073	566 (103)	0.40 (−1.37, 2.16)	0.66	2.29 (0.48, 4.09)	0.0008
AHEI score (raw), linear trend			0.03 (−0.02, 0.09)	0.21	0.10 (0.04, 0.15)	0.0004
AHEI score (per SD), linear trend			0.47 (−0.26, 1.19)		1.35 (0.61, 2.10)	
			[5]Pairs Matching			
AHEI T1	25969	1.43 (0.62)	Reference		Reference	
AHEI T2	25920	1.46 (0.62)	0.03 (0.02, 0.04)	<0.0001	0.03 (0.02, 0.04)	<0.0001
AHEI T3	25864	1.47 (0.62)	0.04 (0.03, 0.05)	<0.0001	0.04 (0.03, 0.05)	<0.0001
AHEI score (raw), linear trend			0.001 (0.001, 0.002)	<0.0001	0.0013 (0.0010, 0.0017)	<0.0001
AHEI score (per SD), linear trend			0.018 (0.014, 0.022)		0.018 (0.013, 0.022)	
			[4]Symbol Digit Substitution			
AHEI T1	15277	19.0 (4.8)	Reference		Reference	
AHEI T2	15540	18.7 (4.8)	−0.12 (−0.23, 0.02)	0.02	−0.24 (−0.34 −0.14)	<0.0001
AHEI T3	15807	18.6 (4.8)	−0.24 (−0.35, −0.14)	<0.0001	−0.44 (−0.54, −0.34)	<0.0001
AHEI score (raw), linear trend			−0.007 (−0.01, −0.004)	<0.0001	−0.014 (−0.017, −0.010)	<0.0001
AHEI score (per SD), linear trend			−0.10 (−0.14, −0.06)		−0.19 (−0.24, −0.15)	
			[5]Trail A			
AHEI T1	13555	3.63 (0.31)	Reference		Reference	
AHEI T2	13505	3.65 (0.31)	0.005 (−0.002, 0.01)	0.14	0.010 (0.003, 0.017)	0.005
AHEI T3	13729	3.66 (0.31)	0.01 (0.003, 0.02)	0.008	0.018 (0.010, 0.025)	<0.0001
AHEI score (raw), linear trend			0.0003 (0.00005, 0.0005)	0.02	0.0005 (0.0003, 0.0008)	<0.0001
AHEI score (per SD), linear trend			0.004 (0.0007, 0.007)		0.007 (0.004, 0.01)	
			[5]Trail B			
AHEI T1	13555	4.17 (0.32)	Reference		Reference	
AHEI T2	13504	4.19 (0.32)	0.007 (−0.0008, 0.01)	0.08	0.016 (0.009, 0.023)	<0.0001
AHEI T3	13729	4.21 (0.32)	0.015 (0.007, 0.022)	<0.0001	0.03 (0.02, 0.04)	<0.0001
AHEI score (raw), linear trend			0.0005 (0.0002, 0.0007)	<0.0001	0.0011 (0.0008, 0.0013)	<0.0001
AHEI score (per SD), linear trend			0.006 (0.003, 0.01)		0.015 (0.012, 0.018)	
			[4]Prospective Memory Test			
	n	% correct	OR (95% CI)	*P*	OR (95% CI)	*P*
AHEI T1	11867	85	Reference		Reference	
AHEI T2	11972	83	0.90 (0.83, 0.96)	0.002	0.88 (0.82, 0.94)	0.0004
AHEI T3	12181	83	0.93 (0.87, 1.00)	0.06	0.90 (0.83, 0.96)	0.003
AHEI score (raw), linear trend			1.00 (0.99, 1.00)	0.04	1.00 (0.99, 1.00)	0.0009
AHEI score (per SD), linear trend			0.97 (0.94, 1.00)		0.95 (0.92, 0.98)	

[1]Model 1: adjusted for age, sex and self−reported race/ethnicity. [2]Model 2: adjusted for age, sex, race/ethnicity, education, Townsend deprivation index, income, employment status, family history of dementia; history of hypertension, diabetes, heart disease, stroke and depression; self−reported health, smoking, physical activity, BMI, fast meal consumption and energy intake. [3]Data are raw mean (SD) scores of each cognitive function test. [4]Positive beta−coefficients for FI (difference in 13−point score) and SDS (difference in number of correct substitutions) and OR >1 for PM (correct on first attempt) correspond to higher performance compared to AHEI tertile 1. [5]Negative beta−coefficients for Pairs

(difference in (log–transformed) number of errors), RT (difference in time, seconds, to respond), Trails A and Trails B (difference in (log transformed) time to complete) correspond to higher performance compared to AHEI tertile 1.

Supplementary Table S10. Cognitive ability and incident dementia (age 55+ years at baseline).

Cognitive Test[3]	Total *n*/cases[3]	All−Cause Dementia			
		Model 1[1]		Model 2[2]	
		HR (95% CI)	*P*	HR (95% CI)	*P*
[4]Fluid Intelligence	35753/362	0.88 (0.83, 0.93)	<0.0001	0.89 (0.84, 0.94)	<0.0001
[5]Reaction Time	78271/834	1.00 (1.00, 1.00)	<0.0001	1.00 (1.00, 1.00)	0.0004
[5]Pairs Matching	77753/819	1.22 (1.09, 1.37)	0.0006	1.21 (1.08, 1.36)	0.001
[4]Symbol Digit Substitution	46625/347	0.87 (0.85, 0.88)	<0.0001	0.87 (0.86, 0.89)	<0.0001
[5]Trail A	40789/265	2.57 (1.81, 3.65)	<0.0001	2.30 (1.62, 3.26)	<0.0001
[5]Trail B	40788/265	6.06 (4.34, 8.46)	<0.0001	5.48 (3.87, 7.75)	<0.0001
[4]Prospective Memory	36020/373	0.65 (0.51, 0.82)	0.0004	0.69 (0.54, 0.87)	0.002

[1]Model 1: adjusted for age, sex and self−reported race/ethnicity. [2]Model 2: adjusted for age, sex, race/ethnicity, education, Townsend deprivation index, income, employment status, family history of dementia; history of hypertension, diabetes, heart disease, stroke and depression; self−reported health, smoking, physical activity, BMI, fast meal consumption and energy intake. [3]Test administered at baseline assessment center or on−line but with follow−up starting from first WebQ (see Methods). [4] OR >1 imply better performance on FI (per 13−point score), SDS (per number of correct substitutions) and PM (correct on first attempt) associate with increased risk. [5] OR >1 imply worse performance on Pairs (per (log−transformed) number of errors), RT (per time, seconds, to respond), Trails A and Trails B (per (log transformed) time to complete) associate with increased risk.

Supplementary Table S11. MIND/AHEI-2010 Adherence and Incident Dementia by Sex[1].

	MIND				AHEI−2010			
Model	Females (*n* = 41877)		Males (*n* = 35521)		Females (*n* = 41877)		Males (*n* = 35521)	
	HR (95% CI)	*P*	HR (95% CI)	*P*	HR (95% CI)	*P*	HR (95% CI)	*P*
	All−Cause Dementia[2]							
T1	Ref		Ref		Ref		Ref	
T2	1.01 (0.79, 1.30)	0.91	1.07 (0.86, 1.33)	0.57	0.84 (065, 1.08)	0.17	1.00 (0.80, 1.25)	0.99
T3	**0.71 (0.54, 0.93)**	**0.01**	1.16 (0.89, 1.51)	0.26	**0.77 (0.60, 0.99)**	**0.04**	1.01 (0.79, 1.30)	0.93
Diet score (raw), linear trend	**0.93 (0.88, 0.98)**	**0.008**	1.05 (0.99, 1.10)	0.11	**0.99 (0.98, 1.00)**	**0.006**	1.00 (0.99, 1.01)	0.80
Diet score (1 SD), linear trend	**0.87 (0.78, 0.96)**		1.09 (0.98, 1.21)		**0.86 (0.77, 0.96)**		1.01 (0.92, 1.12)	
	Alzheimer's Dementia							
T1	Ref		Ref		Ref		Ref	
T2	1.10 (0.75, 1.63)	0.62	0.89 (0.63, 1.26)	0.52	0.78 (0.52, 1.17)	0.23	1.11 (0.78, 1.58)	0.58
T3	0.84 (0.55, 1.28)	0.41	1.15 (0.77, 1.72)	0.49	0.86 (0.59, 1.26)	0.44	1.09 (0.73, 1.60)	0.68
Diet score (raw), linear trend	0.95 (0.87, 1.03)	0.23	1.07 (0.98, 1.16)	0.12	0.99 (0.98, 1.01)	0.28	1.01 (1.00, 1.02)	0.18
Diet score (1 SD), linear trend	0.91 (0.77, 1.06)		1.14 (0.97, 1.34)		0.91 (0.78, 1.08)		1.12 (0.95, 1.31)	

[1]Results from Cox−proportional hazard models adjusted for age, self−reported race/ethnicity, education, Townsend deprivation index, income, employment status, global cognition score, family history of dementia; history of hypertension, diabetes, heart disease, stroke and depression; self−reported health, smoking, physical activity, BMI, fast meal consumption and energy intake. Results in bold-face are statistically significant. [2]*P*=0.008 for MIND×sex and P=0.07 for AHEI×sex.

Supplementary Table S12. Individual MIND components and cognitive health (model 2).

MIND Component	Poor Cognitive Ability							Dementia	
	Fluid intelligence*	Reaction time	Pairs Matching	SDS*	Trail A	Trail B	PM*	All-Cause	Alzheimer's
Higher green leafy intake	+	+	+	+	+	+	+	-	+
Higher other vegetables intake	-	-	-	-	-	-	-	-	-
Higher berry intake	-	+	+	-	+	+	+	-	-
Higher bean intake	-	-	+	+	+	+	+	-	+
Higher whole grains intake	+	+	+	+	+	+	-	-	-
Higher nut intake	+	-	-	-	-	+	-	-	+
Higher fish intake	-	-	-	-	-	-	-	-	-
Olive oil primary cooking oil	+	+	+	+	+	+	+	+	+
Higher poultry intake	-	-	-	-	-	-	-	-	-
Lower butter/margarine intake	+	-	-	-	+	+	+	+	+
Lower cheese intake	+	+	+	+	+	+	+	+	+
Lower processed and red meat intake	+	+	+	+	+	+	+	+	+
Lower fast fried food intake	-	+	+	+	+	+	-	+	+
Lower sweets/pastry intake	+	+	+	+	+	+	+	+	+
Moderate wine intake	-	-	-	-	-	-	-	-	-

*original score estimates (see Table 2) reversed to allow comparison across outcomes. Better cognition/decreased risk of dementia $P < 0.05$ $P < 0.0001$. Poor cognition/increased risk of dementia $P < 0.05$ $P < 0.0001$

Supplementary Table S13. Individual AHEI-2010 components and cognitive health (model 2).

AHEI Component	Poor Cognitive Ability							Dementia	
	Fluid intelligence*	Reaction time	Pairs Matching	SDS*	Trail A	Trail B	PM*	All-Cause	Alzheimer's
Higher vegetables	+	-	+	+	+	+	+	-	-
Higher fruit (not juice)	+	+	+	+	+	+	+	+	+
Higher whole grains	+	+	+	+	+	+	+	-	-
Lower sugar sweetened beverages + fruit juice	+	+	+	+	+	+	+	-	+
Higher nuts & legumes	+	-	+	+	+	+	+	-	+
Lower red meat & products	+	+	+	+	+	+	+	+	-
Lower trans fat	+	-	-	-	+	+	+	-	+
Higher long chain (n-3) fats (EPA +DHA)	-	-	-	-	-	-	-	-	-
Higher PUFA	-	+	-	-	-	-	-	-	-
Lower sodium	-	+	-	-	-	-	-	-	-
Moderate alcohol	-	-	+	-	-	-	-	-	-

*original score estimates (see Table 2) reversed to allow comparison across outcomes. Better cognition/decreased risk of dementia $P < 0.05$ $P < 0.0001$. Poor cognition/increased risk of dementia $P < 0.05$ $P < 0.0001$

Supplementary References

1. McDaniel, M.A.; Scullin, M.K. Implementation intention encoding does not automatize prospective memory responding. *Memory & Cognition* **2010**, *38*, 221–232.
2. Cleutjens, F.A.; Spruit, M.A.; Ponds, R.W.; Dijkstra, J.B.; Franssen, F.M.; Wouters, E.F.; Janssen, D.J. Cognitive functioning in obstructive lung disease: results from the United Kingdom biobank. *Journal of the American Medical Directors Association* **2014**, *15*, 214–219.
3. Lyall, D.M.; Cullen, B.; Allerhand, M.; Smith, D.J.; Mackay, D.; Evans, J.; Anderson, J.; Fawns−Ritchie, C.; McIntosh, A.M.; Deary, I.J. Cognitive test scores in UK Biobank: data reduction in 480,416 participants and longitudinal stability in 20,346 participants. *PloS one* **2016**, *11*, e0154222.
4. Hagenaars, S.P.; Harris, S.E.; Davies, G.; Hill, W.D.; Liewald, D.C.; Ritchie, S.J.; Marioni, R.E.; Fawns−Ritchie, C.; Cullen, B.; Malik, R., et al. Shared genetic aetiology between cognitive functions and physical and mental health in UK Biobank (N=112 151) and 24 GWAS consortia. *Molecular psychiatry* **2016**, *21*, 1624−1632, doi:10.1038/mp.2015.225.
5. UK Biobank Coordinating Centre. Cognitive Function Tests: Assessment Centre. Availabe online: http://biobank.ctsu.ox.ac.uk/crystal/label.cgi?id=100026 (accessed on October).
6. Davies, G.; Lam, M.; Harris, S.E.; Trampush, J.W.; Luciano, M.; Hill, W.D.; Hagenaars, S.P.; Ritchie, S.J.; Marioni, R.E.; Fawns−Ritchie, C. Study of 300,486 individuals identifies 148 independent genetic loci influencing general cognitive function. *Nature communications* **2018**, *9*, 2098.
7. Shen, X.; Cox, S.R.; Adams, M.J.; Howard, D.M.; Lawrie, S.M.; Ritchie, S.J.; Bastin, M.E.; Deary, I.J.; McIntosh, A.M.; Whalley, H.C. Resting−State Connectivity and Its Association With Cognitive Performance, Educational Attainment, and Household Income in the UK Biobank. *Biol Psychiatry Cogn Neurosci Neuroimaging* **2018**, *3*, 878−886, doi:10.1016/j.bpsc.2018.06.007.
8. Cullen, B.; Newby, D.; Lee, D.; Lyall, D.M.; Nevado−Holgado, A.J.; Evans, J.J.; Pell, J.P.; Lovestone, S.; Cavanagh, J. Cross−sectional and longitudinal analyses of outdoor air pollution exposure and cognitive function in UK Biobank. *Scientific reports* **2018**, *8*, 12089.
9. Hill, W.D.; Davies, G.; Harris, S.E.; Hagenaars, S.P.; Liewald, D.C.; Penke, L.; Gale, C.R.; Deary, I.J.; Grp, N.C.W. Molecular genetic aetiology of general cognitive function is enriched in evolutionarily conserved regions. *Translational psychiatry* **2016**, *6*, e980, doi:ARTN e980 10.1038/tp.2016.246.
10. Hagenaars, S.P.; Gale, C.R.; Deary, I.J.; Harris, S.E. Cognitive ability and physical health: a Mendelian randomization study. *Scientific reports* **2017**, *7*, 2651.
11. Nevado−Holgado, A.J.; Kim, C.−H.; Winchester, L.; Gallacher, J.; Lovestone, S. Commonly prescribed drugs associate with cognitive function: a cross−sectional study in UK Biobank. *BMJ open* **2016**, *6*, e012177.
12. Ge, T.; Chen, C.Y.; Doyle, A.E.; Vettermann, R.; Tuominen, L.J.; Holt, D.J.; Sabuncu, M.R.; Smoller, J.W. The Shared Genetic Basis of Educational Attainment and Cerebral Cortical Morphology. *Cereb Cortex* **2019**, *29*, 3471−3481, doi:10.1093/cercor/bhy216.
